# Microbiome structure and function in parallel full-scale aerobic granular sludge and activated sludge processes

**DOI:** 10.1007/s00253-024-13165-8

**Published:** 2024-05-13

**Authors:** Jennifer Ekholm, Frank Persson, Mark de Blois, Oskar Modin, David J. I. Gustavsson, Mario Pronk, Mark C. M. van Loosdrecht, Britt-Marie Wilén

**Affiliations:** 1 Division of Water Environment Technology, Department of Architecture and Civil Engineering, Chalmers University of Technology, Sven Hultins Gata 6, 41296 Gothenburg, Sweden; 2 H2OLAND, Grindgatan 1, 44136 Alingsås, Sweden; 3 Sweden Water Research AB, Ideon Science Park, Scheelevägen 15, 22370 Lund, Sweden; 4 VA SYD, P.O. Box 191, 20121 Malmö, Sweden; 5 Department of Biotechnology, Delft University of Technology, Van Der Maasweg 9, 2629 HZ Delft, The Netherlands

**Keywords:** Aerobic granular sludge, Conventional activated sludge, Full scale, Municipal wastewater treatment, Microbial community, Diversity

## Abstract

**Abstract:**

Aerobic granular sludge (AGS) and conventional activated sludge (CAS) are two different biological wastewater treatment processes. AGS consists of self-immobilised microorganisms that are transformed into spherical biofilms, whereas CAS has floccular sludge of lower density. In this study, we investigated the treatment performance and microbiome dynamics of two full-scale AGS reactors and a parallel CAS system at a municipal WWTP in Sweden. Both systems produced low effluent concentrations, with some fluctuations in phosphate and nitrate mainly due to variations in organic substrate availability. The microbial diversity was slightly higher in the AGS, with different dynamics in the microbiome over time. Seasonal periodicity was observed in both sludge types, with a larger shift in the CAS microbiome compared to the AGS. Groups important for reactor function, such as ammonia-oxidising bacteria (AOB), nitrite-oxidising bacteria (NOB), polyphosphate-accumulating organisms (PAOs) and glycogen-accumulating organisms (GAOs), followed similar trends in both systems, with higher relative abundances of PAOs and GAOs in the AGS. However, microbial composition and dynamics differed between the two systems at the genus level. For instance, among PAOs, *Tetrasphaera* was more prevalent in the AGS, while *Dechloromonas* was more common in the CAS. Among NOB, *Ca*. Nitrotoga had a higher relative abundance in the AGS, while *Nitrospira* was the main nitrifier in the CAS. Furthermore, network analysis revealed the clustering of the various genera within the guilds to modules with different temporal patterns, suggesting functional redundancy in both AGS and CAS.

**Key points:**

• *Microbial community succession in parallel full-scale aerobic granular sludge (AGS) and conventional activated sludge (CAS) processes*.

• *Higher periodicity in microbial community structure in CAS compared to in AGS*.

• *Similar functional groups between AGS and CAS but different composition and dynamics at genus level*.

**Graphical abstract:**

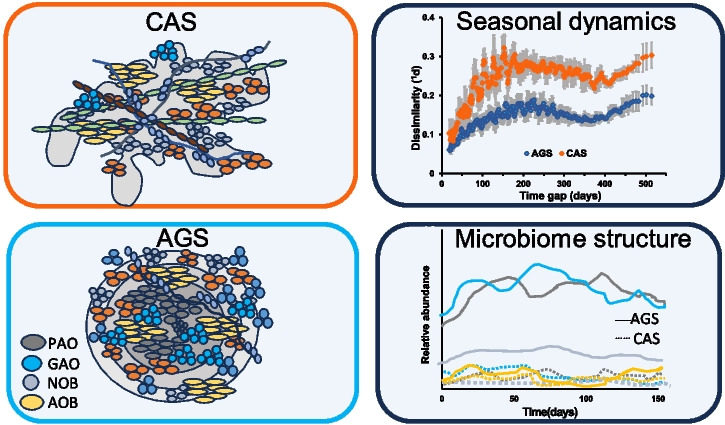

**Supplementary Information:**

The online version contains supplementary material available at 10.1007/s00253-024-13165-8.

## Introduction

Human population growth and increased demand for sustainable water management drive the progress of wastewater treatment systems. Wastewater treatment plants (WWTPs) with high treatment efficiency, small footprint and low energy demand are desired. The conventional activated sludge (CAS) process is widely applied for wastewater treatment but has large land area requirements mainly due to the need for large settlers for the separation of treated water and sludge (van Loosdrecht & Brdjanovic 2014). The CAS process is typically operated in continuously stirred tank reactors (CSTR) and continuous-flow mode. The biomass consists of flocculent sludge, which is kept in the system by recirculation from the settler to the beginning of the CSTR (Henze et al. 2008). Aerobic granular sludge (AGS) is a treatment technology which reduces the land requirements by 25–75% (Pronk et al. 2017). The biomass configuration of AGS is a type of free-floating biofilm (without carriers) with smaller amounts of flocculent sludge. Typically, sequencing batch reactors (SBRs) are used for AGS, where the influent is fed in plug-flow from the bottom of the reactor with simultaneous effluent withdrawal from the top. The feast-famine operation with bottom-fed reactors is selected for fast-settling aggregates (de Kreuk & van Loosdrecht 2004).

The typical functional groups are ammonia-oxidising bacteria (AOB), nitrite-oxidising bacteria (NOB), polyphosphate-accumulating organisms (PAOs), glycogen-accumulating organisms (GAOs), denitrifiers and ordinary heterotrophic organisms. It has previously been shown that activated sludge processes in WWTPs at different locations, even on different continents, have certain species/members within functional groups in common (Begmatov et al. 2022; de Celis et al. 2022; Saunders et al. 2016; Wu et al. 2019). Those members were identified as a core community distinctly coupled to functional traits of the treatment process. Seemingly, environmental and operational conditions are selected for similar core species at a global level (Wu et al. 2019), even though the overall microbiome structure varies between the WWTPs.

Immigration of microorganisms from the influent wastewater has been suggested to strongly control the CAS microbiome (Dottorini et al. 2021), whereas in AGS, immigration seems to be of less importance (Ali et al. 2019; Ekholm et al. 2022). This might be explained by the differences in biomass configuration and solids retention time (SRT). Influent bacteria may more easily incorporate in the flocculent structure compared to the granular biofilm due to the differences in EPS structure (Lin et al. 2013) and availability of surface. Large granules can have an SRT of 140 days (Ali et al. 2019), which is very long compared to the activated sludge processes, which can have an SRT as short as three days. However, differently sized microbial aggregates, such as flocs, small granules and large granules, coexist in full-scale AGS (Ekholm et al. 2022), which results in a spectrum of SRTs (Ali et al. 2019; van Dijk 2022). Furthermore, granules have an internal gradient of SRTs for different microbial populations along the granule radius (Winkler et al. 2012). This probably depends on the exposure to shear forces that lead to more detachment of biomass from the outer layers, where growth is also faster due to the available substrate and space.

Suspended-activated sludge flocs may have a higher abundance of fast-growing microorganisms compared to AGS, as granular biofilms support the survival of slow growers (Wu & Yin 2020). A high growth rate at conditions with high substrate availability is called r-strategy, while a low growth rate and high substrate affinity is called K-strategy, and the ratio between r- and K-strategists is probably different for an SBR and a CSTR (Yin et al. 2022). Thus, different mechanisms are likely influencing the ecological processes within the microbiome in AGS and CAS. Furthermore, the longer and more varying SRT in AGS probably leads to higher microbial diversity than in CAS, which has a shorter and more homogenous SRT. Higher gene cluster diversity was observed in AGS compared with CAS (Burzio et al. 2022). This was, however, not the case in a study comparing full-scale activated sludge with an AGS pilot at the same WWTP (Winkler et al. 2013). Both systems are likely influenced by temperature; observations of variations in alpha and beta diversity in activated sludge were suggested to be largely driven by temperature (Griffin & Wells 2017). Similarly, findings from full-scale AGS microbiome structures indicated that changes in the composition were influenced by seasonal variations in environmental conditions, such as fluctuating temperature (Ekholm et al. 2022).

In comparison with CAS, the knowledge of the microbiome structure of full-scale AGS is still limited. Studies of the complex microbial communities in wastewater treatment plants provide a more detailed understanding of the treatment processes and what factors influence the microbiome assembly and treatment performance over time (Griffin & Wells 2017; Ju & Zhang 2015). A long-term study of the differences in composition, diversity and dynamics of functional groups in parallel CAS and AGS operated at similar conditions has not yet been carried out. In this paper, the process performance and microbiome structure in an SBR-AGS process and a CAS system were studied for 1.5 years. The plant operation enabled comparison between two parallel AGS reactors as well as with the parallel CAS reactor. The microbiome structure was examined to shed light on the assembly processes during seasonal dynamics in temperature, flow and nutrient loads, coupled with treatment performance.

## Materials and methods

### Plant, operation and influent wastewater

**Fig. 1:**
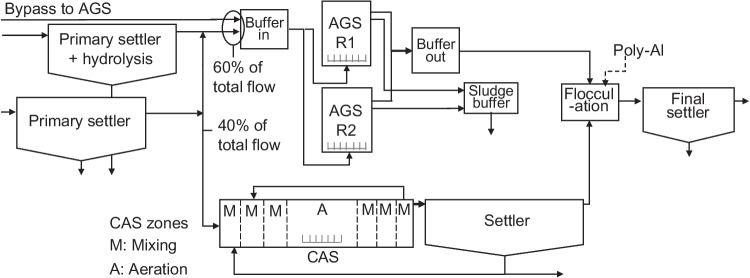
Schematic view of the biological processes at Österröd WWTP (not to scale)

The study of the parallel AGS and CAS processes was performed at the Österröd WWTP, Strömstad, Sweden (58°55′59.1″N 11°11′48.8″E). The wastewater is first treated by inlet screens (6 mm), an aerated fat- and sand trap and two primary settlers, followed by the biological treatment process (Fig. 1) consisting of two AGS Nereda® reactors (Nereda® is trademark of Royal HaskoningDHV) and a parallel CAS reactor, treating approximately 60% and 40% of the incoming wastewater, respectively (Supplementary Fig. S1). Part of the flow to the AGS bypasses the primary settlers. Furthermore, hydrolysis/fermentation is applied in the primary settler feeding the AGS. During the study, the total flow to the AGS was mainly coming from the primary setter with hydrolysis, and the bypassed flow was, on average, 20% ± 16%. The effluents from the AGS and the CAS are mixed in a flocculation tank (dosage of poly-aluminium chloride as precipitation chemical) and lead to a final settler. The rejected water from the dewatering of the sludge led to the primary settler or the activated sludge reactor. The start-up of the AGS process during the first 16 months of operation (June 2018 to October 2019) was previously described with respect to granulation, microbial community dynamics and process performance (Ekholm et al. 2022). At the time of this study, the AGS had been in operation for two years and the CAS for one year. The reactors were studied during stable operation from June 2020 to December 2021.

The treatment plant received increased inflow during rain events due to the combined sewer system, and occasionally seawater intrusion occurred. The region of the WWTP is heavily subjected to summer tourism, leading to a large increase in the organic and nutrient concentrations and loads (Supplementary Table S1 and Fig. S2). The effluent quality demands are 10 mg L^−1^ of BOD_7_, 70 mg L^−1^ of COD, 0.30 mg L^−1^ of phosphorus and 15 mg L^−1^ of nitrogen.

The AGS reactors are operated as SBRs with a volume of 758 m^3^ each and plug-flow bottom feeding. The reaction phase typically consists of main aeration, with (when remaining cycle time allows) pulse-aeration for pre- and/or post-denitrification, including stripping of nitrogen gas and settling, followed by simultaneous decanting and feeding. The AGS reactors are equipped with sensors and analysers for temperature, redox potential, water level and concentrations of dissolved oxygen (DO), nitrate, ammonium and phosphate (Endress Hauser). The DO set-point was 2 mg/L during dry weather flow and 3 mg/L during wet weather flow. During high flows, due to rainy weather, the cycle time is shortened in order to treat the increased flow.

The CAS reactor (1300 m^3^) consists of seven zones, where typically zone 4 (520 m^3^) was aerated, and zones 1–3 and 5–7 were mixed without aeration. This creates anoxic conditions for both pre- and post-denitrification. No external carbon source was supplied. The anoxic volumes were normally 470 m^3^ (zones 1–3) and 310 m^3^ (zones 5–7). The influent wastewater and the return sludge were directed to zone 1. From February 19th, 2021, the nitrate recirculation flow was pumped to zone 2 and, before that, to zone 1, which created anoxic feeding conditions rather than anaerobic. The CAS reactor is equipped with sensors for DO, ammonium and nitrate. The operational parameters are presented in Table 1 and Supplementary Fig. S3.

**Table 1:** Operational parameters of the AGS and CAS reactors, average ± standard deviation, from October 2020 to September 2021

Parameter	Unit	AGS1	AGS2	CAS
Solids retention time (SRT)^a^	d	28–49	28–66	26–43
Influent flow	m^3^ d^−1^	1280 ± 570	1280 ± 600	1680 ± 660
Sludge concentration^a^	g MLSS L^−1^	8.8 ± 1	9.1 ± 1	3.1 ± 0.8
Biomass ratio^a^	VSS/MLSS	0.86 ± 0.02	0.86 ± 0.02	0.86 ± 0.04
SVI_10_/SVI_30_^a^	ratio	1.0 ± 0.05	1.0 ± 0.04	1.6 ± 0.35
Influent pH	-	8.2 ± 1.1	8.2 ± 1.1	-
F/M-ratio^b^	kg BOD_7_ (kg MLSS d)^−1^	0.018 ± 0.008	0.018 ± 0.007	0.024 ± 0.007
P.E. load^c^	P.E	1650	1650	1500
Return sludge flow	m^3^ d^−1^	-	-	2174 ± 1277
Exchange ratio	-	0.46 ± 0.06	0.45 ± 0.06	-
Feed velocity	m h^−1^	3.44 ± 0.02	3.44 ± 0.05	-
Hydraulic retention time	h (min–max)	14 (10–26)	14 (10–27)	16 (11–32); (8^d^ (5^d^–16^d^)

^a^Average SRT based on averages ± Std. For the AGS, the different aggregate sizes have different SRTs. Sampling from June 2020 to December 2021

^b^Based on kg BOD_7_ divided by the total amount of sludge in the reactor

^c^Based on average BOD_7_ load and 70 g BOD_7_ per person and day

^d^Values do not include the secondary settler after the CAS reactor

### Influent wastewater characteristics

The average influent concentrations are presented in Supplementary Fig. S4 and Table S1. Two actions were undertaken to increase the concentration of organic matter in the influent to the AGS: hydrolysis in the primary settler (Fig. 1) by increasing the sludge level and partly bypassing the primary settler (Supplementary Fig. S1). These measures resulted in increased influent concentrations of BOD_7_, COD and SS to the AGS compared with the CAS.

### Reactor loads

The volumetric loads of BOD_7_ were fluctuating (Supplementary Fig. S2), probably depending on the removal efficiency of suspended solids over the pre-settler and also due to hydrolysis and bypassed flow to the AGS (Supplementary Table S2). The loads of TN and TP increased during the summer and were generally higher on the AGS than on the CAS due to the flow division (60/40%). The biomass-specific load of BOD_7_ was rather similar on the two systems; however, those of TN and TP were often higher on the CAS compared with the AGS (Supplementary Fig. S5). In the calculations of the loads, the settling time was included for the AGS but not for the CAS.

### Sample collection and water analyses

Samples of the AGS were taken monthly at depths of 1.5 m and 5 m with a Ruttner sampler (approximately 1.5 L) during the main aeration phase, whereafter the sludges from the two depths were mixed, and a biomass sample of 15 mL was collected. Activated sludge samples of 15 mL were taken from the aerated zone on a monthly basis.

Samples of influent and effluent were taken as flow-proportional water samples collected from June 2020 to October 2021. The influent and effluent water samples were analysed for BOD_7_, COD, ammonium, nitrate, nitrite, TKN, TN, phosphate and TP according to standard methods (APHA 1992). The soluble BOD_7_ and phosphate were analysed after filtration with 0.45-µm pore size filters. Analyses of mixed liquor suspended solids (MLSS), volatile suspended solids (VSS), total solids (TS), volatile solids (VS) and sludge volume index (SVI) after 10 min and 30 min were measured according to standard methods (APHA 2005). SVI_30_ of the CAS was measured by mixing 40% sludge with 60% effluent. The distribution of granule sizes was analysed by sieving 1 L of sludge through sieves with pore sizes of 2 mm, 1.4 mm, 0.6 mm, 0.4 mm and 0.2 mm. The sample remaining on each sieve was washed, collected and dried at 105 °C for TS measurements. The sludge morphology was observed by light microscopy (Olympus BX53) with micrographs taken by a digital camera (Olympus DP11).

### DNA extraction, sequencing and bioinformatics

The DNA extraction, PCR amplification, purification, sequencing and bioinformatics were performed as previously described (Ekholm et al. 2022). Briefly, sludge biomass was centrifuged (3000 × g, 5 min), and the remaining pellets were kept and stored at − 20 °C. The reactor sludge samples were thawed and homogenised using a BagMixer 100 Minimix (Interscience), whereafter DNA was extracted from 350 µL of homogenised sludge using the FastDNA spin kit for soil (MP Biomedicals). The V4 region of the 16S rRNA gene was amplified by PCR, and sequencing of the amplicons was conducted on a MiSeq (2 × 300) using reagent kit V3 (Illumina). A total of 41,776 to 148,5104 sequence reads were obtained per sample. Two bioinformatic pipelines (sequence processing and generation of count tables) were used, DADA2 v.1.16 (Callahan et al. 2016) and VSEARCH v.2.13.1 (Rognes et al. 2016), and the results were merged, resulting in one consensus count table of amplicon sequence variants (ASVs) created in the bioinformatics package qdiv (Modin et al. 2020). The taxonomic assignment was done with Midas 4 (Dueholm et al. 2022). The consensus count table was rarefied to the lowest number of reads (41,776) by subsampling without replacement. Multivariate statistics and visualisations were generated in qdiv. Raw sequence reads are deposited at the NCBI sequence read archives (SRA), accession PRJNA952867.

### Statistical methods

Paired sample T-test was used to analyse differences between reactors, and statistical significance was considered for *p*-values < 0.05. Wilcoxon signed-rank test was used when the residuals were non normal distributed. For correlation analysis, Spearman’s correlation test was used.

Network analysis was carried out for each reactor separately. Only ASVs that were present in at least two samples and had a maximum relative abundance exceeding 0.05% were included. Pairwise correlations between ASVs were calculated using FastSpar v1.0.0 (Watts et al. 2018; Friedman and Alm 2017) and Spearman’s ρ. For FastSpar, *p*-values were calculated based on 1000 random permutations of the original data, and correlations with *p* < 0.05 were kept for further analysis. For Spearman’s ρ, the Benjamini–Hochberg procedure for setting the false discovery rate at 0.05 was used to sort out significant correlations. Pairwise correlations that were statistically significant and had a correlation coefficient > 0.5 with both FastSpar and Spearman’s ρ were used in the network analysis, which was done using NetworkX v3.1.

## Results

### Process performance

**Fig. 2:**
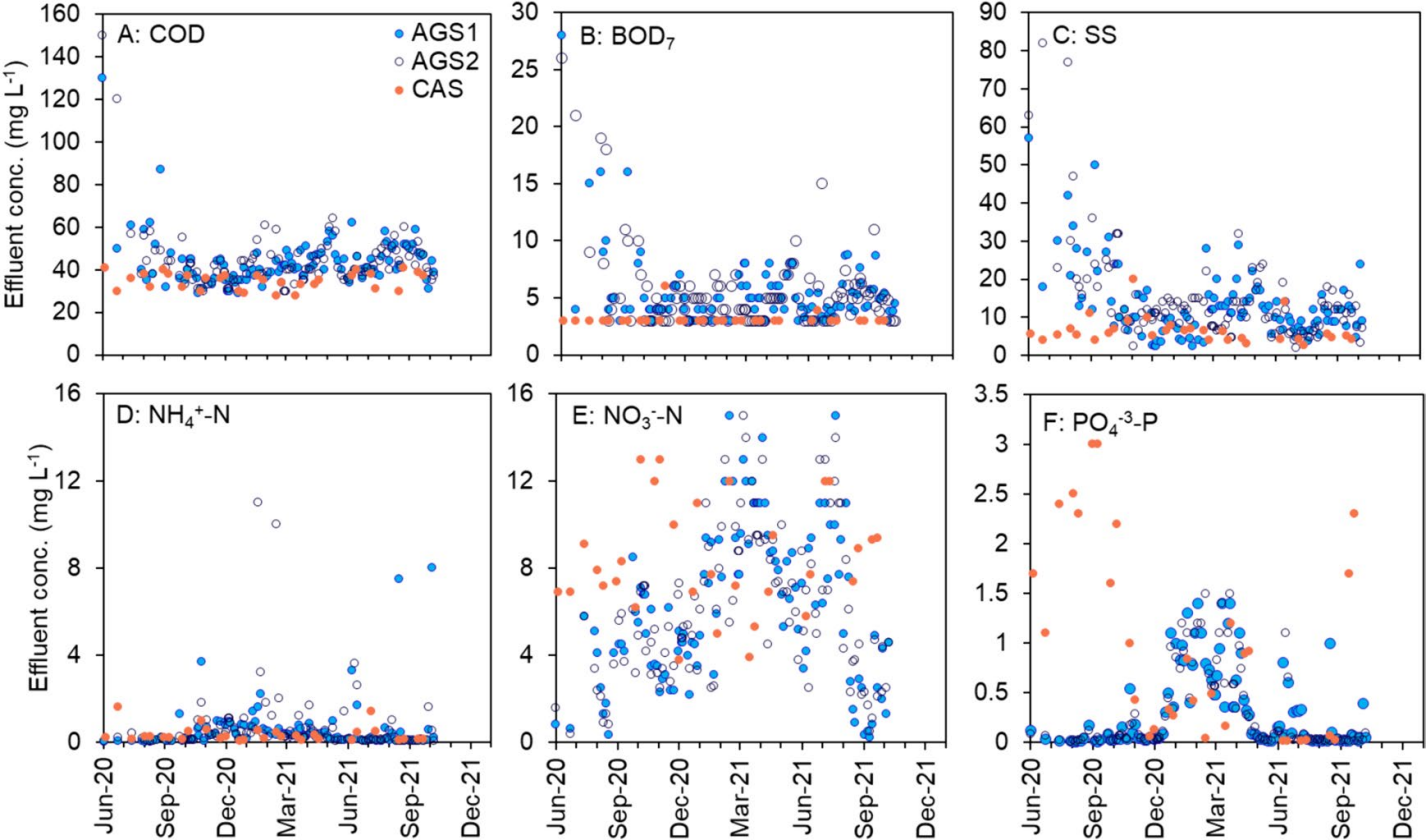
Effluent concentrations of (**A**) COD, (**B**) BOD_7_, (**C**) SS, (**D**) NH_4_^+^-N, (**E**) NO_3_^−^-N and (**F**) PO_4_^−^.^3^-P from AGS1, AGS2 and CAS. Note that the influent concentrations were different in the two systems (Supplementary Table S1 and Fig. S4)

The effluent concentrations of organic matter, nitrogen and phosphorous from the two AGS reactors (Fig. 2) were similar (Supplementary Table S3, *p* > 0.05). The average total BOD_7_ and COD concentrations from the AGS were generally low, on average 5 ± 2 mg L^−1^ and 42 ± 7 mg L^−1^, respectively. The average ammonium and nitrite concentrations were 0.7 ± 1.0 mg L^−1^ and 0.1 ± 0.1 mg L^−1^, respectively. Both phosphate and nitrate effluent concentrations were fluctuating and peaked in the winter 2020/2021 and early spring 2021. During the same time, the influent concentration of BOD_7_ was low, with occasionally low BOD_7_/TN (1.6–4.5) and BOD_7_/TP ratios (21–63) (Supplementary Fig. S4). The effluent concentration of BOD_7_ from the CAS was normally < 3 mg L^−1^ (shown as 3 mg L^−1^, which was the detection limit in Fig. 2). The ammonium concentration was, on average, 0.3 ± 0.3 mg L^−1^, and the effluent SS was, on average, 7 ± 4 mg L^−1^. The TP and phosphate effluent concentrations from the CAS were fluctuating and peaked in September 2020, March 2021 and September 2021. The effluent concentration of nitrate was fluctuating throughout the investigated period (Fig. 2E).

**Fig. 3:**
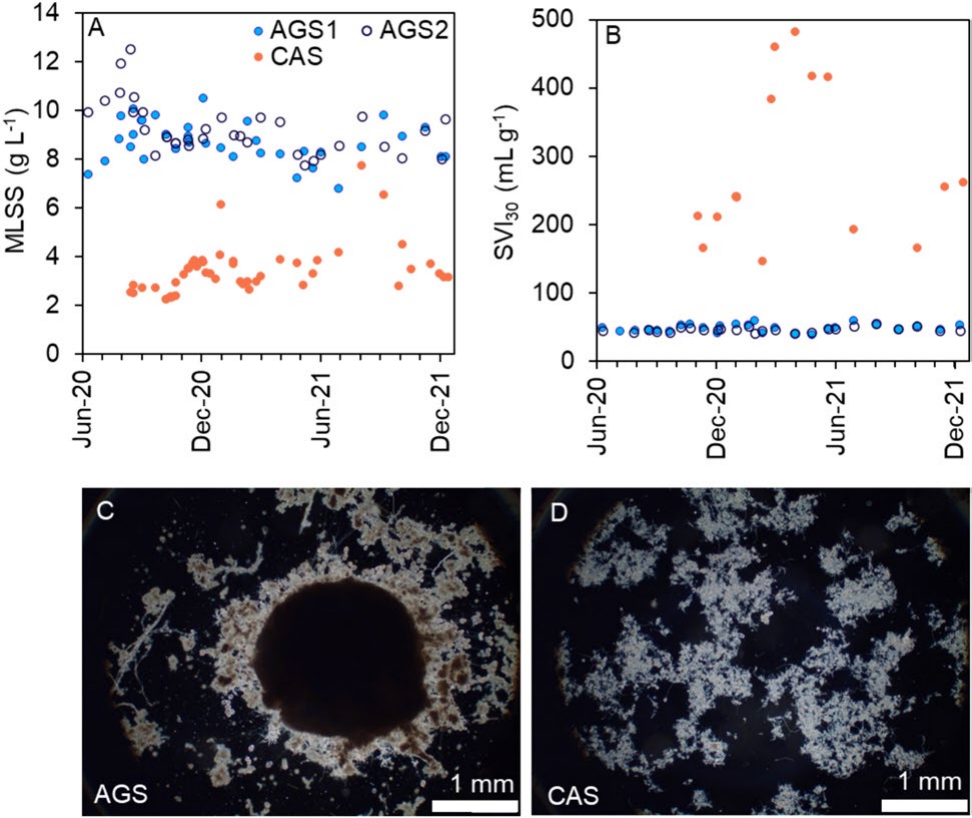
(**A**) Sludge concentrations as MLSS, (**B**) settling properties as SVI_30_ and microscope pictures of (**C**) AGS and (**D**) CAS. The scale bar represents 1 mm

The effluent concentrations of TP, phosphate and nitrate were lower from the AGS than the CAS, whereas the BOD_7_, COD, SS and ammonium concentrations were lower from the CAS (Supplementary Table S4, *p* < 0.05). The large difference in effluent COD between AGS and CAS was mainly caused by the difference in SS. This might be related to the AGS technology and/or to the higher content of SS in the influent to the AGS system (Supplementary Table S1). The biomass-specific removal rates were similar in the AGS and the CAS, with higher rates during the summers (Supplementary Fig. S6) when the loads were higher (Supplementary Fig. S5). However, the AGS had higher volumetric removal rates than the CAS (Supplementary Fig. S7) due to the differences in biomass concentration (Fig. 3A).

### Sludge characteristics

The sludge concentrations in the AGS reactors were 8–10 g L^−1^, and in the CAS, they were approximately 3.1 g L^−1^ (Fig. 3A). The sludge in the AGS had excellent settling properties with SVI_30_ of approximately 50 mL g^−1^, whereas the settling properties (SVI_30_) in the CAS varied from 150 to 500 mL g^−1^ (Fig. 3B). The granule size distribution was dominated by large granules (> 2 mm) in both AGS reactors (Supplementary Fig. S8). The TS-fraction of flocculent sludge (< 200 µm) was 0.19 ± 0.07 in AGS1 and 0.16 ± 0.06 in AGS2.

### Microbial community dynamics

Hill numbers (Jost 2006) with diversity order *q* were applied to measure alpha diversity (^*q*^*D*). All ASVs are given equal weight irrespective of their relative abundances at *q* = 0, and the index is equivalent to species richness. For *q* = 1, the relative abundances of the ASVs are considered, and the index can be interpreted as the number of ‘common’ ASVs. Dissimilarity (^*q*^*d*) between communities was also measured based on the Hill number framework (Chao et al. 2014).

#### Alpha diversity

**Fig. 4:**
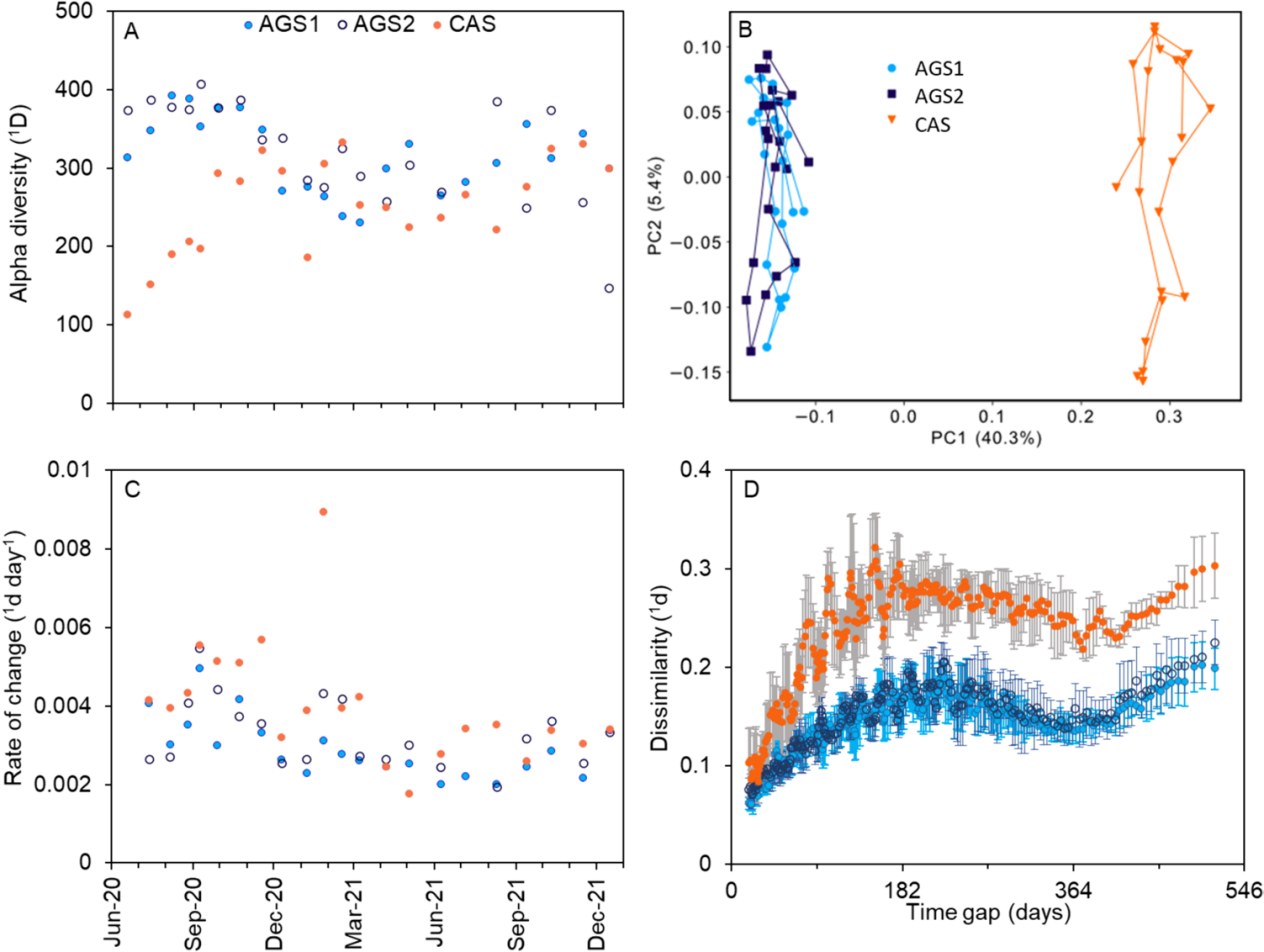
(**A**) Alpha diversity (*q* = 1), (**B**) principal coordinate analysis (PCoA) of the AGS reactors and CAS (*q* = 1), (**C**) rate of change in dissimilarity per day (*q* = 1) and (**D**) dissimilarity (*q* = 1) as a function of the time gap between biomass microbial communities in the CAS, AGS1 and AGS2. A moving window average of 10 samples is represented at each point; error bars show the standard deviation

The alpha diversity (*q* = 0 and *q* = 1) varied over time and was slightly higher in the AGS compared with the CAS (*p* < 0.05) (Fig. 4A, Supplementary Fig. S9). Alpha diversity at both *q* = 0 and *q* = 1 increased with temperature in the AGS reactors (*p* < 0.05) but not in the CAS, where the diversity at *q* = 1 decreased with increasing temperature (*p* < 0.05) (Supplementary Fig. S10).

#### Dissimilarity (beta diversity)

The community structure was clearly separated between the microbiomes in the AGS reactors and the CAS and evolved over time (Fig. 4B). Pairwise comparisons of samples taken the same day from the CAS and AGS reactors indicated stable dissimilarities between CAS and AGS (^1^*d* = 0.46 ± 0.030), while the parallel AGS reactors were similar to each other (^1^*d* = 0.094 ± 0.020) (Supplementary Fig. S11). The rate of change (dissimilarity divided by days between consecutive samples) was higher in the CAS than the AGS reactors at *q* = 1 (*p* < 0.05) but was similar between the systems at *q* = 0 (*p* > 0.05) (Fig. 4C, Supplementary Fig. S12). Furthermore, the temporal changes in the microbial community composition expressed as a function of the time gap between two samples with averaging over ten values in a moving window were assessed as dissimilarities (*q* = 1). Periodicity was observed in both AGS and CAS with maximum dissimilarities at a time gap of around half a year (Fig. 4D).

#### Microbial community networks

**Fig. 5:**
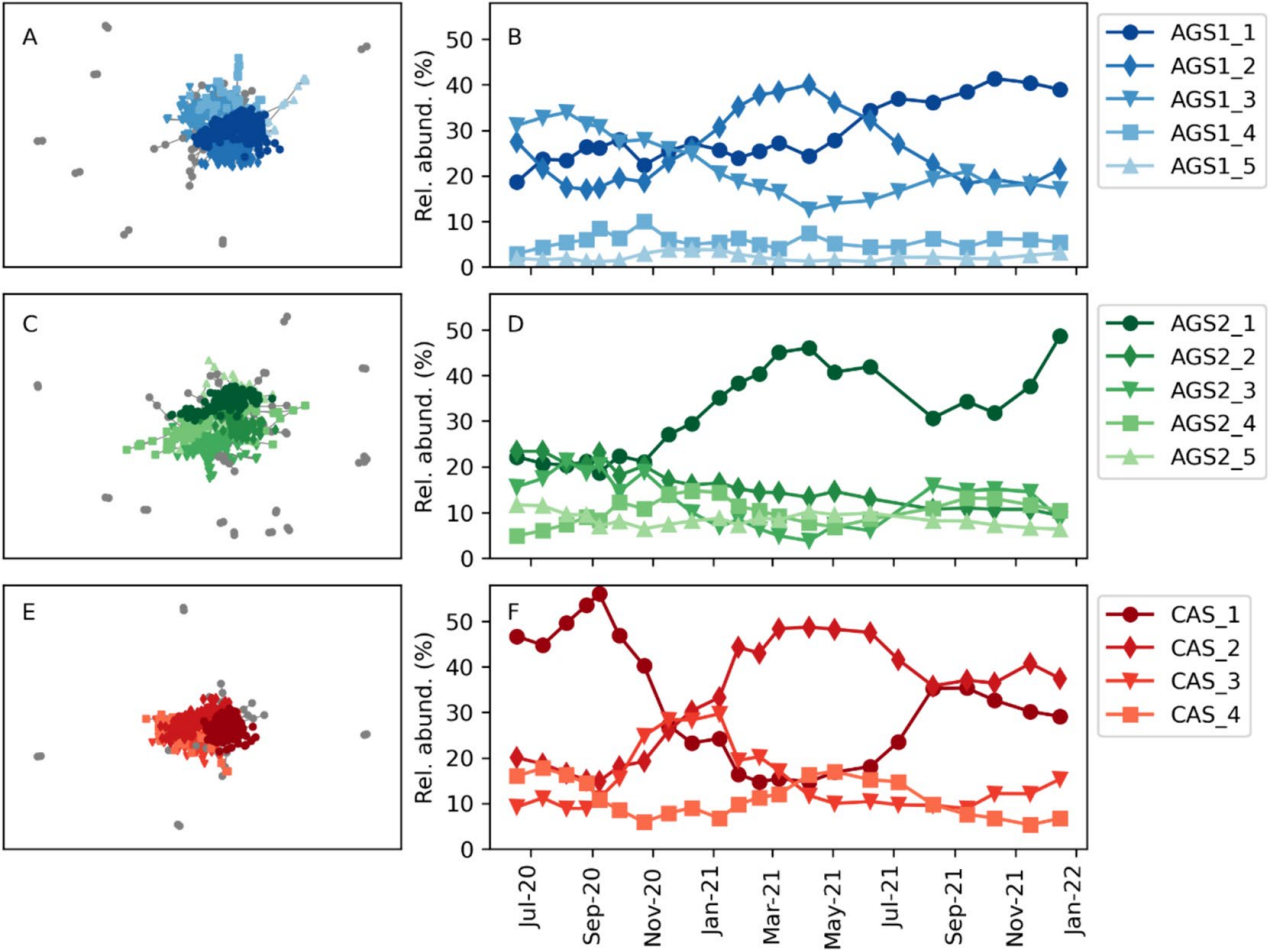
Microbial community networks of positive correlations in AGS1 (**A**, **B**), AGS2 (**C**, **D**) and CAS (**E**, **F**). The networks are shown in **A**, **C** and **E**. The relative abundances of major modules are shown in **B**, **D** and **F**

Positive correlations between ASVs were visualised in network analysis, one for each reactor (AGS1, AGS2 and CAS, Fig. 5). Some fundamental differences in network structure could be observed. The CAS had a larger number of co-occurring ASVs that were interconnected in the network (745 nodes, 6334 edges) than the two AGS reactors (632 nodes and 3621 edges in AGS1; 586 nodes and 2802 edges in AGS2). The AGS reactors, on the other hand, had higher modularity (0.60 in AGS1 and 0.62 in AGS2) than the CAS (0.53). Modularity indicates whether the network can be subdivided into smaller clusters of nodes. Five major network modules, including at least 10 ASVs and making up a total relative abundance of at least 2% in a sample, were identified in each AGS and four in the CAS. The modules had different temporal patterns, either showing seasonal periodicity or increasing/decreasing trends over time. The distribution of ASVs (within main guilds) between the modules is shown in Supplementary Table S6.

#### Microbial community composition and functional guilds

**Fig. 6:**
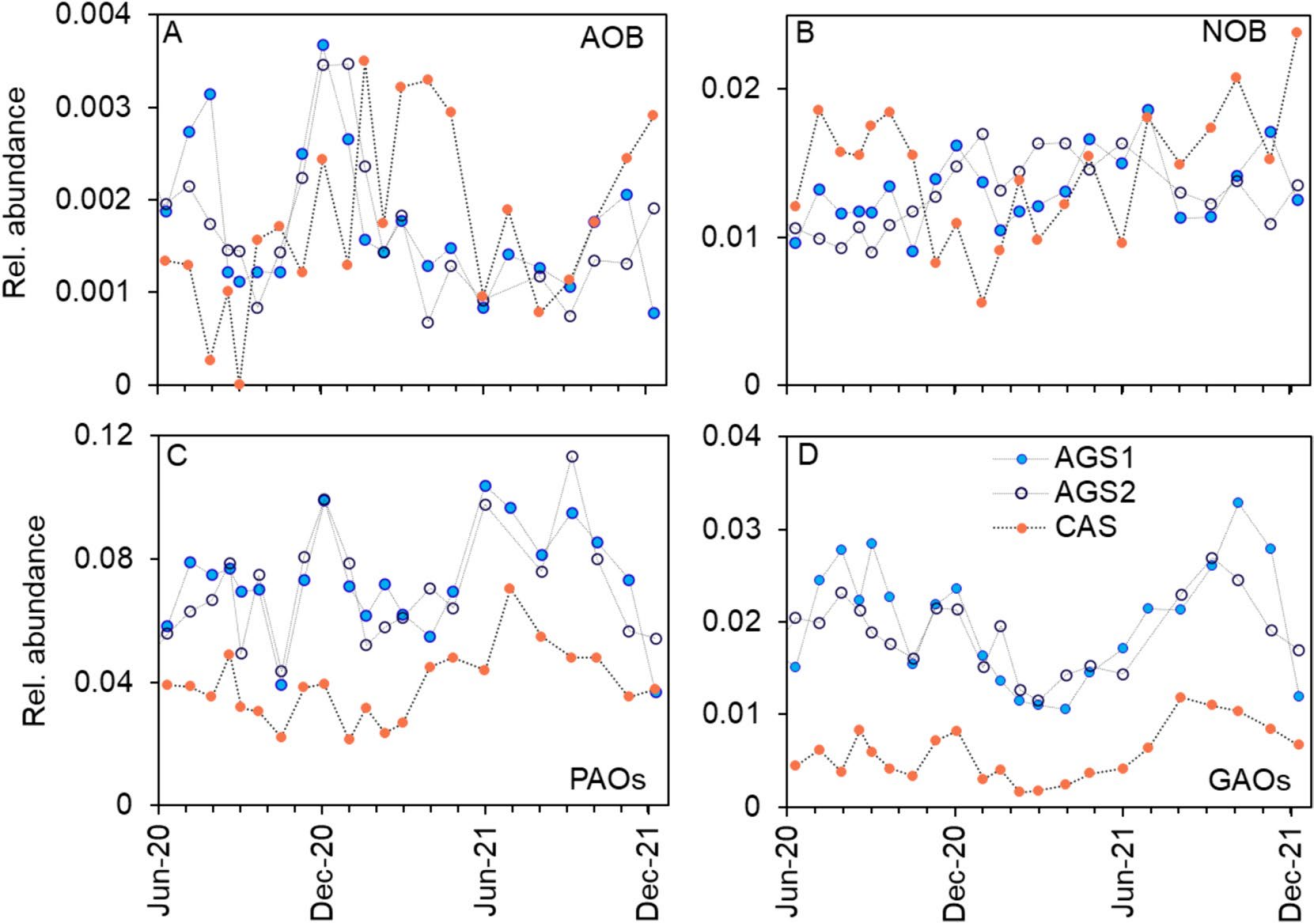
Relative abundance of the functional groups: (**A**) AOB, (**B**) NOB, (**C**) PAOs and (**D**) GAOs in AGS1, AGS2 and CAS

**Fig. 7:**
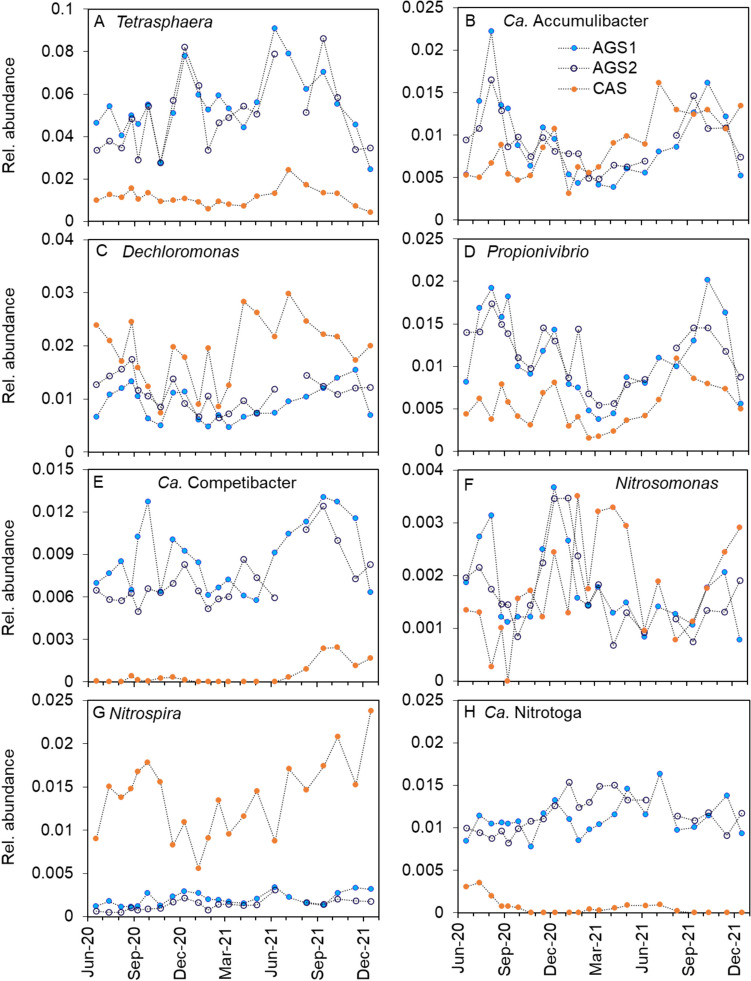
Relative abundances in AGS1, AGS2 and CAS of the genera: (**A**) *Tetrasphaera*, (**B**) *Ca.* Accumulibacter, (**C**) *Dechloromonas*, (D) *Propionivibrio*, (**E**) *Ca*. Competibacter, (**F**) *Nitrosomonas*, (**G**) *Nitrospira* and (**H**) *Ca*. Nitrotoga

The relative abundances of many of the 20 most abundant genera were pronouncedly different between the AGS reactors and the CAS (Supplementary Fig. S13). The PAOs and GAOs were considerably more abundant in the AGS than in the CAS (Fig. 6). *Tetrasphaera* was the main PAO in the AGS, represented by two abundant ASVs, while the abundance in the CAS was much lower (Supplementary Fig. S13). *Ca*. Accumulibacter had a similar distribution in both systems, while *Dechloromonas* was more abundant in the CAS (Fig. 7B,C). The three PAOs had different correlations with temperature in the two systems. *Tetrasphaera* increased in relative abundance at higher temperatures in the CAS but not in the AGS (Supplementary Table S5 and Fig. S15). In contrast, *Ca*. Accumulibacter and *Dechloromonas* were positively correlated with temperature in the AGS but not in the CAS. Among the GAOs, both *Ca*. Competibacter and *Propionivibrio* were more abundant in the AGS than the CAS (Fig. 7D,F, Supplementary Fig. S16). The difference was particularly large for *Ca*. Competibacter, which was barely detected in the CAS. The abundance of *Propionivibrio* was furthermore positively correlated with temperature in both the AGS and CAS (Supplementary Table S5 and Fig. S17).

The nitrifying populations were small, with different dynamics in the AGS and the CAS, although the average abundances of AOB and NOB were comparable between the systems (Fig. 6, Supplementary Fig. S18). The microbial communities in AGS and CAS harboured populations of *Nitrosomonas* (AOB), *Ca*. Nitrotoga (NOB) and *Nitrospira* (NOB or comammox (Daims et al. 2015; van Kessel et al. 2015)). The relative abundance of *Ca*. Nitrotoga was pronouncedly higher in the AGS, whereas *Nitrospira* was higher in abundance in the CAS (Fig. 7G, H). *Nitrosomonas* was observed in higher diversity in the CAS (four ASVs) than in the AGS (two ASVs) (Supplementary Fig. S18). Additionally, *Nitrosomonas* showed signs of species replacement in the CAS where ASV384 was disappearing with time, while ASV1215 was appearing. There were a few correlations between the relative abundances of the nitrifying populations and temperature (Supplementary Table S5 and Fig. S19). For instance, *Nitrosomonas* increased in abundance at lower temperatures in the CAS but not the AGS.

Among putative denitrifiers, *Rhodoferax, Zoogloea, Paracoccus* (mainly in the CAS) and *Thauera* (mainly AGS) were detected (Dueholm et al. 2022), as well as the aforementioned *Tetrasphaera*, *Ca.* Accumulibacter, *Dechloromonas* and *Ca.* Competibacter, which contains clades capable of denitrification (Gao et al. 2019; Marques et al. 2018; Petriglieri et al. 2021; Wang et al. 2021). However, a functional group of denitrifiers is hard to specify, as many microorganisms carry genes for full or truncated denitrification (Gao et al. 2019).

Among the aerobic heterotrophs, for example, *Rhodoferax, Terrimonas*, *Flavobacterium*, *Ferruginibacter* and OLB12 within the family *Bacteroidota* were common in both the AGS and CAS. In line with the observations of high SVI, the filamentous genus *Microthrix* was common in the CAS, but considerably less abundant in the AGS reactors (Supplementary Figs. S13 and S20).

#### Correlations between ASVs in functional guilds

The PAOs, GAOs, AOB and NOB all contained multiple ASVs that could be found in several network modules. The PAOs contained 24 ASVs that could be assigned to a network module. All four major modules in the CAS, three in AGS1 and four in AGS2, contained PAOs. The GAOs contained 11 ASVs, the AOB contained four ASVs, and the NOB contained five ASVs that could be assigned to a module. Also, among these functional guilds, ASVs were found in several of the major network modules (Supplementary Table S6).

## Discussion

### Microbial community assembly

#### Higher diversity in AGS, coupled with differences in substrate gradients

The results showed slightly higher species richness in the AGS compared with the CAS (Supplementary Fig. S9), which could be attributed to the higher probability of different microenvironments in the AGS. Similar observations were made during the start-up of the AGS plant, where AGS1 seeded with activated sludge showed slightly lower species richness compared to AGS2, which was seeded with granular sludge (Ekholm et al. 2022). In the AGS, granular biofilm and flocs form different niches (Ali et al. 2019), whereas only flocs form the habitats in the CAS. The granular biofilm structure enables steep concentration gradients of substrates and DO (de Kreuk et al. 2007), which is different from the flocculent biomass. Furthermore, larger granules will have a higher probability of being present at the bottom of the reactor (van Dijk et al. 2020), leading to a heterogenous distribution along the reactor depth, thereby creating microenvironments with different conditions during the feeding through the bed of settled biomass (van Dijk 2022). Therefore, the granule biofilm with its spatial gradients and the feast-famine (temporal gradients) condition probably supports both r- and K-strategists (Wu & Yin 2020). In the CAS, the flocs within one zone of the reactor likely have a similar availability for the substrate, as the biomass and substrates are expected to be homogeneously distributed. These conditions would lead to a smaller variety of r- and K-strategists in the CAS. Moreover, the spectrum of SRTs in the AGS in various aggregate sizes (Ali et al. 2019; van Dijk 2022) probably promoted microbial diversity. Previous research comparing full-scale activated sludge with an AGS pilot plant at the same WWTP found, however, comparable diversity between the two systems despite the difference in biomass configuration (Winkler et al. 2013). The difference in the results of Winkler et al. (2013) compared to our findings might depend on the larger set of samples and the higher resolution of the more recent techniques used in this study. Amplicon sequencing, which is used in this study, compared to DGGE used by Winkler et al. (2013), gives a much more detailed analysis of the microbial community (Rastogi & Sani 2011).

The diversity was increasing with the temperature in the AGS (Supplementary Fig. S10B). At higher temperatures, the consumption rates of organic matter and nutrients would increase, resulting in steeper gradients of substrates. This may have prompted the growth of microorganisms with a larger variety of substrate preferences distributed along the granule depth. Different mechanisms seem to have shaped the diversity in the CAS, which showed no or weak negative correlation with temperature (*q* = 1, *p* < 0.05) (Supplementary Fig. S10). Besides lacking pronounced substrate gradients, microbial communities in flocs, as opposed to granules (Ekholm et al. 2022), are to a large extent affected by immigration (Ali et al. 2019; Dottorini et al. 2021), which may have contributed to the observed results. This also confirms earlier findings in full-scale CAS, showing that temperature plays a minor role in controlling diversity (Ju & Zhang 2015). Previous studies of microbial community assembly in activated sludge show contradictory results, both that the temperature may have little impact on the diversity (Ju & Zhang 2015), and that there is a seasonal influence of temperature (Griffin & Wells 2017).

#### Succession of the microbial communities

The similarity in diversity and community composition over time of AGS1 and AGS2 suggests that the reactor conditions were shaping the communities in a similar manner (Fig. 4, Supplementary Fig. S11). Reactor type was clearly more important than seasonal differences for shaping the microbial communities, as visualised by PCoA (Fig. 4B), where the first axis separating AGS and CAS explained a larger share of the variation than the second axis separating samples over time. The rate of change of the microbial communities in all reactors suggests continuous succession, with a higher rate of change (*q* = 1) observed in the flocculent sludge (Fig. 4C). Flocs typically have shorter SRT than large granules (Ali et al. 2019). Furthermore, flocs are influenced by immigration from the influent community and, hence, are more exposed to seasonal fluctuations (de Celis et al. 2022; Griffin & Wells 2017; Sun et al. 2021).

The periodicity in the microbial community structure of both AGS and CAS (Fig. 4D) suggests that the seasonal environmental conditions were influencing the assembly of the microorganisms. This periodicity was different in the two systems, with higher dissimilarities in the CAS reactor due to the higher rate of change. Because of the relatively shorter SRT in the CAS (compared with large granules), the microbial community in the CAS reactor will likely have more co-variation or dependence on environmental fluctuations (Griffin & Wells 2017).

The dissimilarity in community composition (*q* = 1) between the AGS and the CAS over the entire time series (Supplementary Fig. S11) indicates that the reactor conditions applied in the AGS (SBRs with anaerobic feeding in plug-flow and selective sludge to maintain granules) were indeed selecting for a different community than in the CAS (CSTR with extensive sludge recirculation). The biofilm structure matrix likely also enabled both cooperation and competition, which created conditions for different microbes to grow (Fig. 3C,D) (Nadell et al. 2016). However, the feed composition differed due to the bypass of influent, resulting in higher concentrations of BOD_7_, COD and SS to the AGS, which likely also contributed to the dissimilarity of the microbial community in comparison with the CAS. The organic substrate diffusibility was previously shown to influence the microbial community composition in lab-scale AGS (Layer et al. 2019).

### Functional groups

#### Substrate availability was a key factor for the PAOs

Both reactor systems performed biological P removal, albeit at a higher extent in the AGS (Fig. 2). The relative abundances of PAOs were higher in the AGS, probably due to the higher influent concentrations of organic substrate and the anaerobic feeding regime. *Tetrasphaera* was the most abundant genus in the AGS (Fig. 7A), which might reflect the influent composition of the bypassed flow with increased complexity. *Tetrasphaera* is commonly detected in municipal wastewater treatment, a fermenting PAO able to utilise complex substrates such as amino acids and sugars (Nielsen et al. 2019), whereas *Ca*. Accumulibacter, which prefers volatile fatty acids (VFAs), was detected in lower abundance in the AGS (Fig. 6B).

PAOs and GAOs were detected in higher abundances in the AGS compared to the CAS (Fig. 6), likely dependent on the difference in operation regime (batch-wise plug-flow versus continuous flow), influent concentrations of organic matter, biomass configuration and SRT. The general trends in the dynamics of the sum of genera within the groups of PAOs and GAOs over time were similar in the AGS and CAS (Fig. 6), suggesting that environmental conditions in both processes influenced these functional groups in a similar manner. However, the abundant genera within PAOs and GAOs differed in abundance between the CAS and the AGS (Supplementary Figs. S14 and S16). At the level of individual ASVs, dynamics were different between the CAS and AGS, suggesting that the reactor conditions favoured different ASVs under various periods in the CAS and the AGS (Supplementary Figs. S14, S16, S18). Previously, during the start-up of the AGS plant, the PAOs within *Tetrasphaera* and *Ca.* Accumulibacter were found to be localised along the entire depth of the granules (Ekholm et al. 2022).

*Microthrix* was detected in higher abundances in the CAS reactor (Supplementary Fig. S20) and can accumulate P, but so far, the typical P-cycling has not been confirmed (Nierychlo et al. 2021). Nevertheless, the ability to accumulate P suggests that *Microthrix* might have contributed to the removal of P in the CAS. *Microthrix* is known to cause troublesome bulking sludge in activated sludge processes (Nierychlo et al. 2021; Wang et al. 2019) and was probably contributing to the relatively high SVI of the activated sludge (Fig. 3B). The difference in abundance between the AGS and the CAS suggests that *Microthrix* was outcompeted in the granular microbial community. Similar results were observed during the upgrading of a full-scale activated sludge process to AGS, which resulted in a shift in the microbial community composition from filamentous bacteria in the flocculent sludge to EPS-producing bacteria in the granular sludge (Świątczak & Cydzik-Kwiatkowska 2018).

Elevated effluent concentrations of phosphate in both processes (Fig. 2F) were probably caused by a lack of organic substrate. Low influent concentrations of bioavailable organic matter during the winter were likely caused by limited hydrolysis in the sewer system (AGS and CAS) and in the pre-settler (AGS) (Supplementary Fig. S4). For the AGS, the bypass was turned off occasionally in the summer of 2021, which contributed to the lower organic substrate. In the CAS, also limited anaerobic conditions in space and time were likely factors for elevated phosphate concentrations.

#### The biomass configuration influenced the AOB and NOB community composition

Nitrification was observed in both systems with low effluent concentrations of ammonium (Fig. 2D) and nitrite. The relative abundances of AOB and NOB were generally similar in the AGS and the CAS (Fig. 6), which corresponds to the general similarity of the nitrogen concentrations in both the influent and effluent. However, the composition varied considerably, with a higher abundance of *Ca*. Nitrotoga in the AGS, while *Nitrospira* was more common in the CAS. The aggregate types (granules versus flocs, Fig. 3C,D) may explain the distribution differences. In a recent study, *Ca*. Nitrotoga had five times higher abundance in the core than the outer parts of granules (Cydzik-Kwiatkowska et al. 2022), suggesting that the higher abundance in the AGS might be related to beneficial conditions created at depth in the granules. Furthermore, *Ca*. Nitrotoga has been detected in higher abundances in large granules, whereas *Nitrospira* proliferated in the smallest sludge fraction (< 200 µm) in an anammox system (Liu et al. 2017). Genomic profiling suggested that some species of *Ca*. Nitrotoga have abilities to grow in low-oxygen environments (Boddicker and Mosier 2018). The diffusion resistance might create microaerophilic conditions in the granular core for the benefit of *Ca*. Nitrotoga, while substrate gradients in small granules and flocs are less pronounced. However, it has been demonstrated that *Nitrospira* can grow in a wide range of DO and suggested that the *Nitrospira* lineages have different oxygen affinity (Latocheski et al. 2022). *Nitrosomonas* was more diverse in the numbers of ASVs in the CAS compared to the AGS (Supplementary Fig. S18), which may be associated with higher availability of space, oxygen and ammonium in sludge flocs compared to granules. Moreover, the relative abundance of *Nitrosomonas* in the CAS was negatively correlated with the temperature, which might be related to the higher DO concentrations (3 mg L^−1^) applied during cold temperatures (March 2021, Supplementary Fig. S3C). Previous studies suggest that AOB and NOB have a lower affinity for oxygen than ordinary aerobic heterotrophs (Cho et al. 2022). The higher DO during winter in the CAS probably resulted in higher competitiveness of *Nitrosomonas*, resulting in increased relative abundance. In our previous study, AOB within *Nitrosomonas* and NOB within *Ca.* Nitrotoga were found to be more abundant at the surface, with some colonies in the deeper parts of the granules (Ekholm et al. 2022).

Many microorganisms with the ability to denitrify were detected in various relative abundances in the two systems, possibly including denitrifying PAOs and GAOs. Despite the presence of denitrifiers, the effluent concentrations of nitrate were fluctuating in both systems. This was likely caused by low influent BOD/N and BOD/P-ratios (Supplementary Fig. S4), varying activity of denitrifying PAOs and GAOs, and competition for organic carbon with non-denitrifying members of the PAOs and GAOs during feeding and with other aerobic heterotrophs in the aeration phase.

#### Functional redundancy ensured sustained reactor function over time

The network analysis showed that ASVs belonging to the same functional guild (i.e. PAO, GAO, AOB or NOB) were distributed between different modules (Fig. 5). This suggested they belonged to different ‘subcommunities’, which had different temporal dynamics in the reactors. The distribution of ASVs from the same functional guild in different modules may suggest functional redundancy. Changes in environmental conditions, such as temperature and biotic interactions, vary over time and could affect the fitness of different species carrying out the same metabolic function in the reactor (Louca et al. 2018). Such differences in fitness create functional redundancy and make the reactor functions resilient because if changes in conditions lead to the decline of one species, another species within the same functional guild but with higher fitness for the new reactor condition would take over. The somewhat higher modularity in the AGS in comparison with the CAS may be a consequence of a larger number of ecological niches within the granules.

Microbial community succession and process performance were studied in two full-scale AGS reactors and one CAS for 1.5 years. Both processes performed nitrification, denitrification and enhanced biological phosphorus removal. Effluent concentrations of N and P were lower in the AGS, while concentrations of SS, COD and BOD_7_ were lower in the CAS. Slightly higher microbial community diversity and modularity in the AGS compared with the CAS was likely due to the multitude of aggregate sizes (both granules and flocs), granular biofilm and feast-famine operation, creating different niches and spatial as well as temporal substrate gradients, which would support a wider range of microorganisms including both r- and K-strategists. Seasonal periodicity was observed at a higher magnitude for the CAS compared to the AGS, as well as a higher rate of change in the microbiome structure and a network consisting of more interconnected nodes, suggesting that the flocculent sludge was co-varying with environmental conditions at a higher extent. Reactor conditions such as anaerobic feeding, bottom-fed plug-flow and selective sludge discharge were shaping the microbial communities in the two AGS reactors in a similar manner and selected for a different community composition compared with the CAS. The two processes had differences in relative abundances of genera within the functional groups, for example *Tetrasphaera*, *Nitrospira* and *Ca*. Nitrotoga. These findings elucidate the different structures of microbial communities in AGS and CAS during seasonal dynamics in temperature, flow and nutrient loads.

## Author contributions (CRediT)

J.E.: conceptualisation, methodology, formal analysis, investigation, writing – original draft, writing – review and editing. F.P.: conceptualisation, methodology, supervision, funding acquisition, writing – review and editing. M.d.B.: conceptualisation, methodology, formal analysis, supervision, funding acquisition, writing – review and editing. O.M.: data curation, writing – review and editing. D.J.I.G.: conceptualisation, methodology, supervision, project administration, funding acquisition, writing – review and editing. M.v.L.: writing – review and editing. M.P.: writing – review and editing. B-M.W.: conceptualisation, methodology, supervision, funding acquisition, writing – review and editing.

## Supplementary Information

Below is the link to the electronic supplementary material.

Supplementary file1 (PDF 2281 KB)

## Data Availability

Raw sequence reads are deposited at the NCBI sequence read archives (SRA), accession PRJNA952867.
