# Supplementary material for: Microbiome structure and function in parallel full-scale aerobic granular sludge and activated sludge processes
Source: Appl Microbiol Biotechnol. 2024 May 13;108:334. doi: 10.1007/s00253-024-13165-8 (PMC11090927; doi:10.1007/s00253-024-13165-8)
Supplement: Supplementary file 1 [file 253_2024_13165_MOESM1_ESM.pdf]

Supplementary Information

Microbiome structure and function in parallel full-scale aerobic granular sludge and activated sludge processes

Jennifer Ekholm [a], Frank Persson [a], Mark de Blois [b], Oskar Modin [a], David J.I. Gustavsson [c,d], Mario Pronk [e], Mark C. M. van Loosdrecht [e], Britt-Marie Wilén [a,*]

[a] Division of Water Environment Technology, Department of Architecture and Civil Engineering, Chalmers University of Technology, Sven Hultins gata 6, SE-41296 Gothenburg, Sweden

[b] H2OLAND, Grindgatan 1, SE-44136 Alingsås, Sweden

[c] Sweden Water Research AB, Ideon Science Park, Scheelevägen 15, SE-22370 Lund, Sweden

[d] VA SYD, P.O. Box 191, SE-20121 Malmö, Sweden

[e] Department of Biotechnology, Delft University of Technology, Van der Maasweg 9, 2629 HZ Delft, The Netherlands

* Corresponding author. Email: britt-marie.wilen@chalmers.se

Tables

Table S1. Influent wastewater composition average ± standard deviation from October 2019 to September 2021, n is the number of samples.

Parameter	AGS Sep-Jun	AGS Jul-Aug	CAS Sep-Jun	CAS Jul-Aug
BOD_7	97 ± 45	160 ± 40	69 ± 27	104 ± 13
$BOD_{7, soluble}$ [a]	30 ± 17	70 ± 20	26 ± 15	53 ± 6
COD	250 ± 100	380 ± 90	187 ± 76	270 ± 44
TP	2.9 ± 1.2	5.0 ± 1.0	2.7 ± 1.2	4.3 ± 0.6
$TP_{, soluble}$ [a]	1.4 ± 0.6	2.6 ± 0.8	1.6 ± 1.0	3.1 ± 0.5
TN	29 ± 13	51 ± 12	26 ± 10	46 ± 6
NH_4^+-N	24 ± 9	45 ± 12	23 ± 9	37 ± 4
SS	130 ± 70	160 ± 60	80 ± 20	80 ± 20
BOD_7 / TN	3.3 ± 1.0	3.3 ± 0.9	2.7 ± 0.7	2.3 ± 0.3
BOD_7 / TP	34 ± 9	32 ± 7	27 ± 6	25 ± 3
n	103	41	28	13

[a] Filtered through 0.45 µm, Eurofins

Table S2. Operation of the AGS hydrolysis and bypass

Operational parameter	Period
Hydrolysis was implemented by increasing sludge bed in pre-settler	31 August 2019
Time for hydrolysis in pre-settler was increased	4 June – 15 July 2021
Recirculation in pre-settler (pump installed)	11 May – 23 June 2020 and 14 January – 24 May 2021
No recirculation in pre-settler	June 2020 – January 2021 and from May 2021 onwards
No bypassed flow to AGS	15 – 17 September 2020<br>29 October – 4 November 2020<br>21–25 January 2021<br>2–16 March 2021<br>2 June–21 July 2021<br>29 July–2 August 2021<br>01 October – 31 December 2021

Table S3. T-test p-values for paired effluent samples from AGS1 and AGS2 (June 2020 to September 2021).

Parameter	BOD_7	COD	SS	TP	PO_4^{3-}-P	NH_4^+-N	NO_3^--N
p-value	0.493353	0.574628	0.051834	0.949995	0.441061	0.657089	0.979494

Table S4. T-test for paired effluent samples from AGS (average of AGS1 and AGS2) and the CAS, p-values and the mean concentrations (mg L^{-1}) from June 2020 to September 2021.

Parameter	BOD_7	COD	COD soluble	SS	TP	PO_4^{3-}-P	NH_4^+-N	NO_3^--N
p-value	0.00086	0.000392	0.000258	0.000273	0.016902	0.004904	0.035835	0.000318
AGS mean	6.3	53	35	17	0.64	0.35	0.95	5.4
CAS mean	3.1	35	32	6	1.2	1.004	0.34	8.4

Table S5. p-values from Spearman's correlation test for relative abundance and temperature.

p-value	*Nitro-spira*	*Ca.* Nitro-toga	*Nitroso-monas*	*Ca.* Accumuli-bacter	*Dechloro-monas*	*Tetra-sphaera*	*Propioni-vibrio*	*Ca*_Competi-bacter
**AGS1**	0.045	0.83	0.40	0.0002	0.012	0.72	0.0003	0.039
**AGS2**	0.11	0.0002	0.31	0.0002	0.0001	0.77	0.003	0.86
**CAS**	0.092	0.020	0.0001	0.89	0.086	0.000003	0.003	0.13
Spearman's coefficient								
**AGS1**	-0.43			0.71	0.53		0.70	0.44
**AGS2**		-0.71		0.72	0.72		0.61	
**CAS**		0.49	-0.73			0.77	0.59	

Table S6. Number of ASVs among PAOs, GAOs, AOB and NOB belonging to each major network module.

**Reactor**	**Module**	**Number of ASVs**	**PAOs**	**GAOs**	**AOB**	**NOB**
AGS1	AGS1_1	170	8	6	0	2
AGS1	AGS1_2	158	3	0	1	1
AGS1	AGS1_3	200	6	3	1	0
AGS1	AGS1_4	42	0	0	0	1
AGS1	AGS1_5	26	0	0	0	0
AGS2	AGS2_1	159	4	2	0	3
AGS2	AGS2_2	96	1	0	0	0
AGS2	AGS2_3	149	3	4	0	1
AGS2	AGS2_4	65	4	2	1	0
AGS2	AGS2_5	60	0	0	1	0
CAS	CAS_1	223	5	2	0	1
CAS	CAS_2	263	6	3	2	0
CAS	CAS_3	147	2	0	0	0
CAS	CAS_4	79	6	0	1	1

Figures

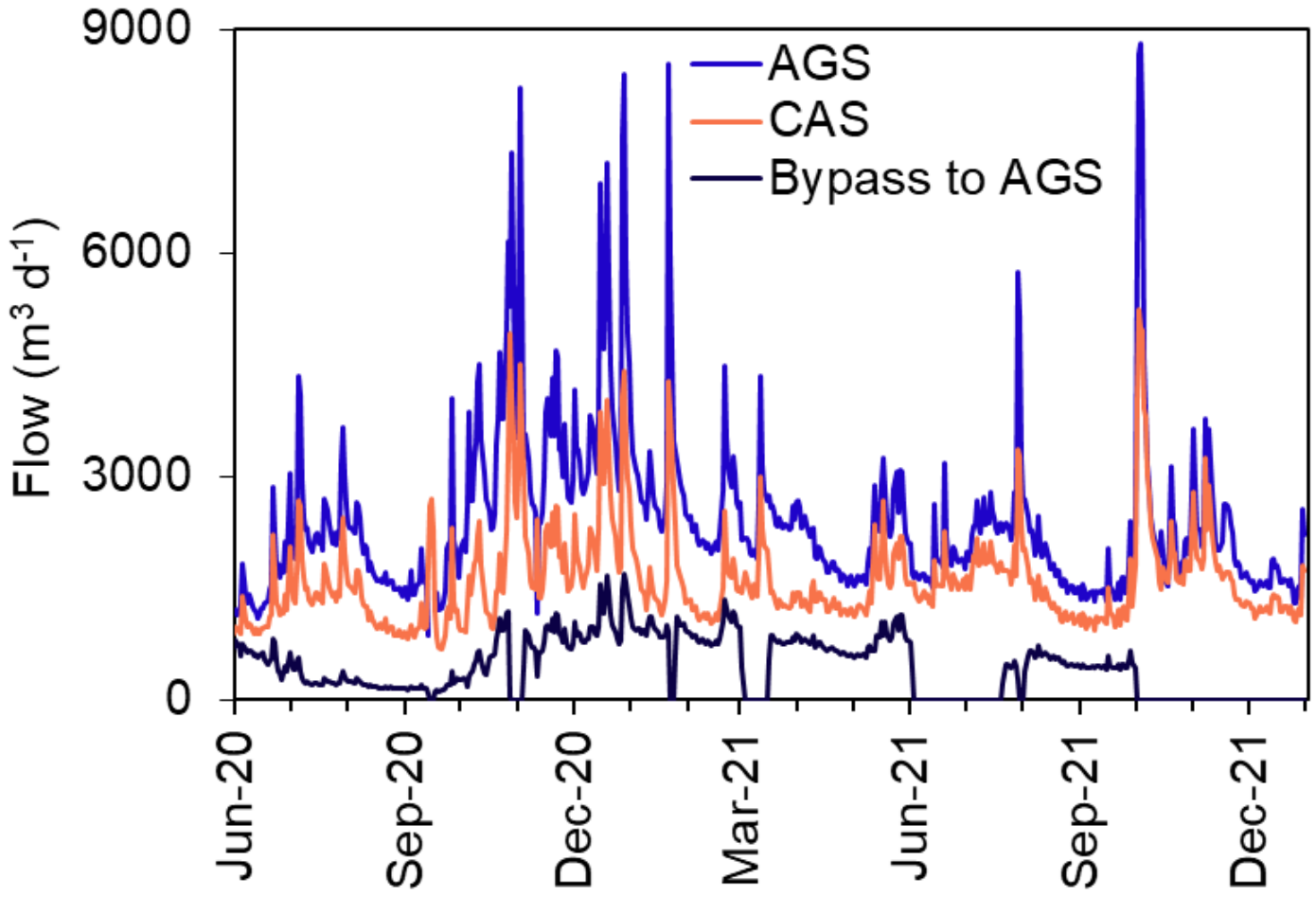

Figure S1. Flow to the AGS, CAS and bypassed flow to the AGS.

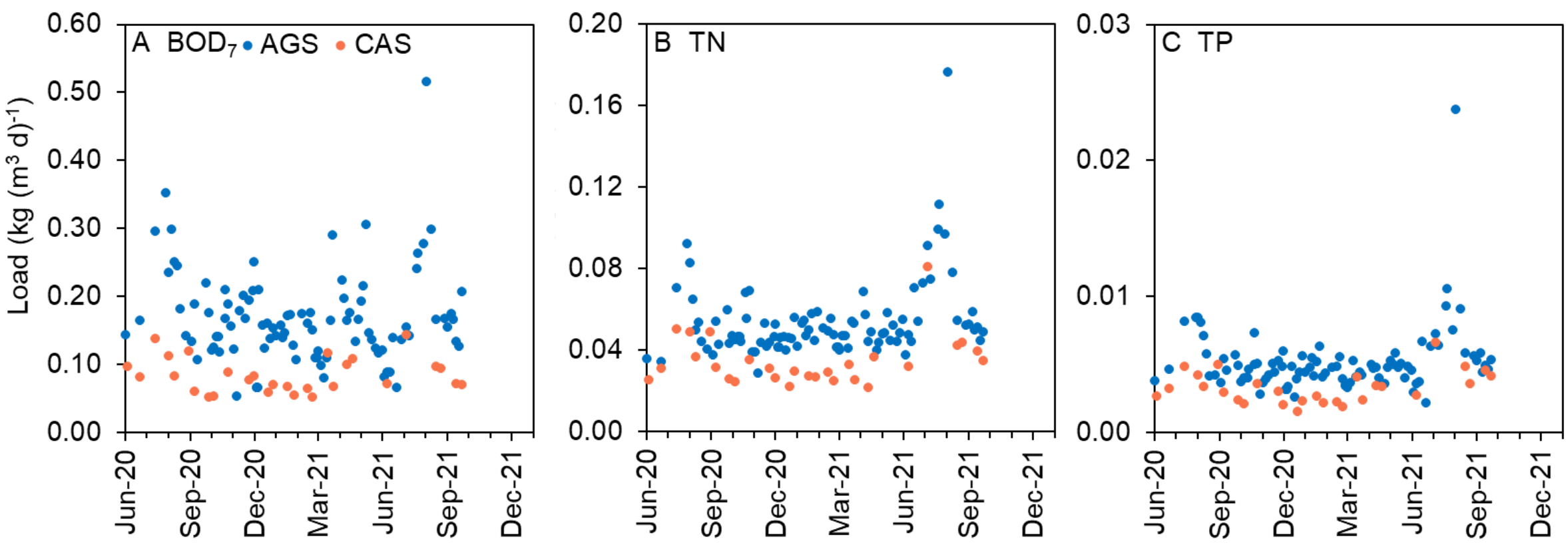

Figure S2. Volumetric load of A) BOD_7, B) TN, C) TP in the AGS and the CAS.

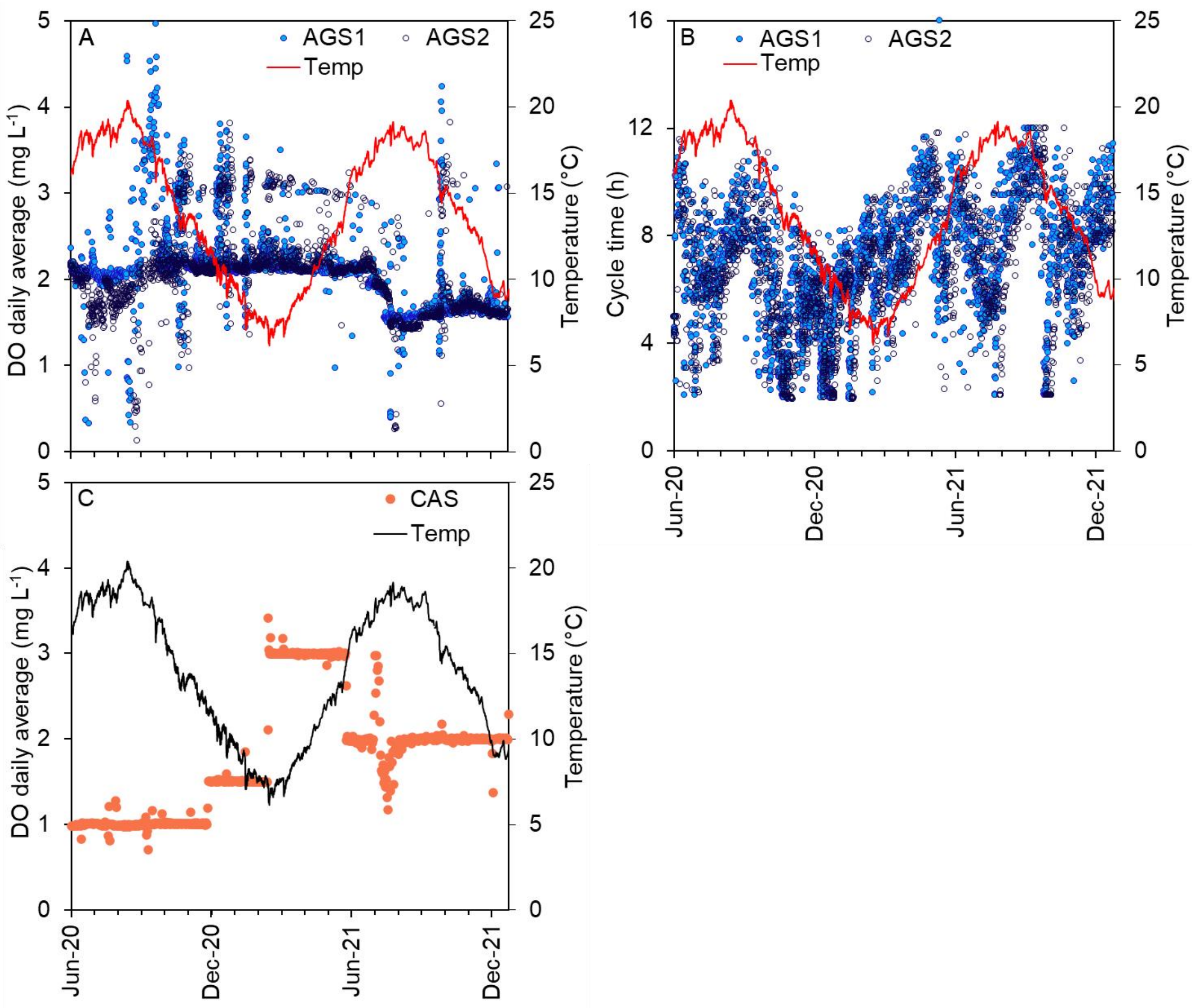

Figure S3. AGS daily average of A) DO and temperature, B) cycle time and temperature, C) CAS daily average DO and temperature (zone 4).

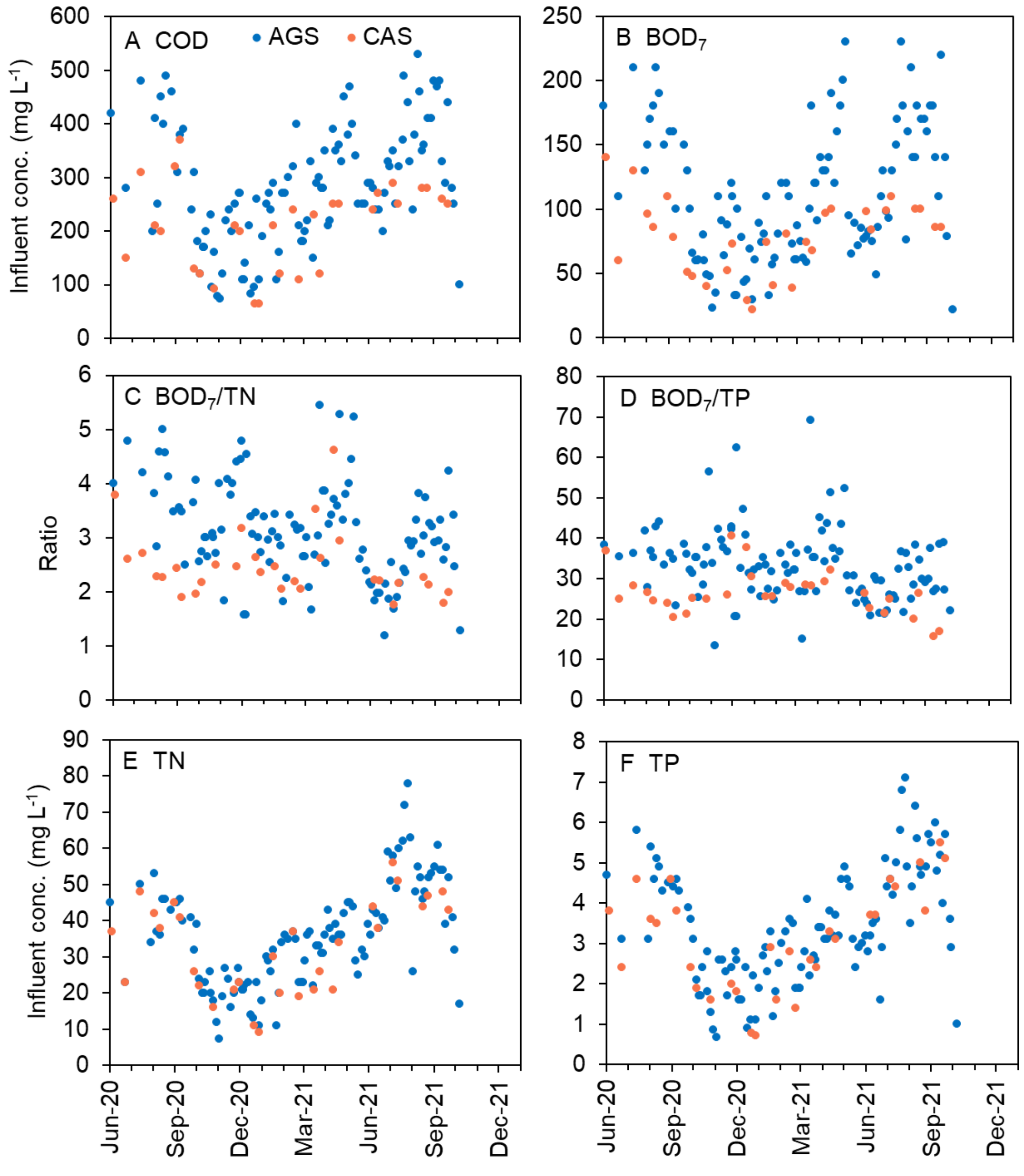

Figure S4. Influent concentrations or ratios of A) COD, B) BOD_7, C) BOD_7/TN, D) BOD_7/TP, E) TN, and F) TP.

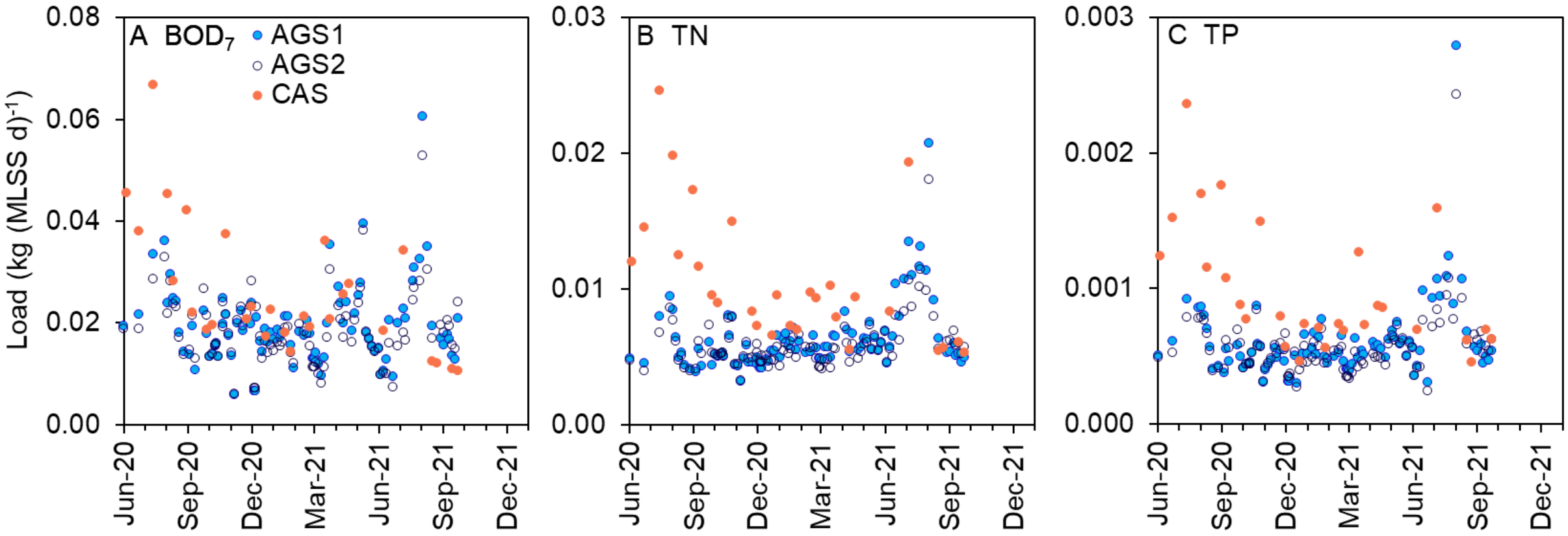

Figure S5. Biomass specific load of A) BOD_7, B) TN, C) TP in AGS1, AGS2, and the CAS.

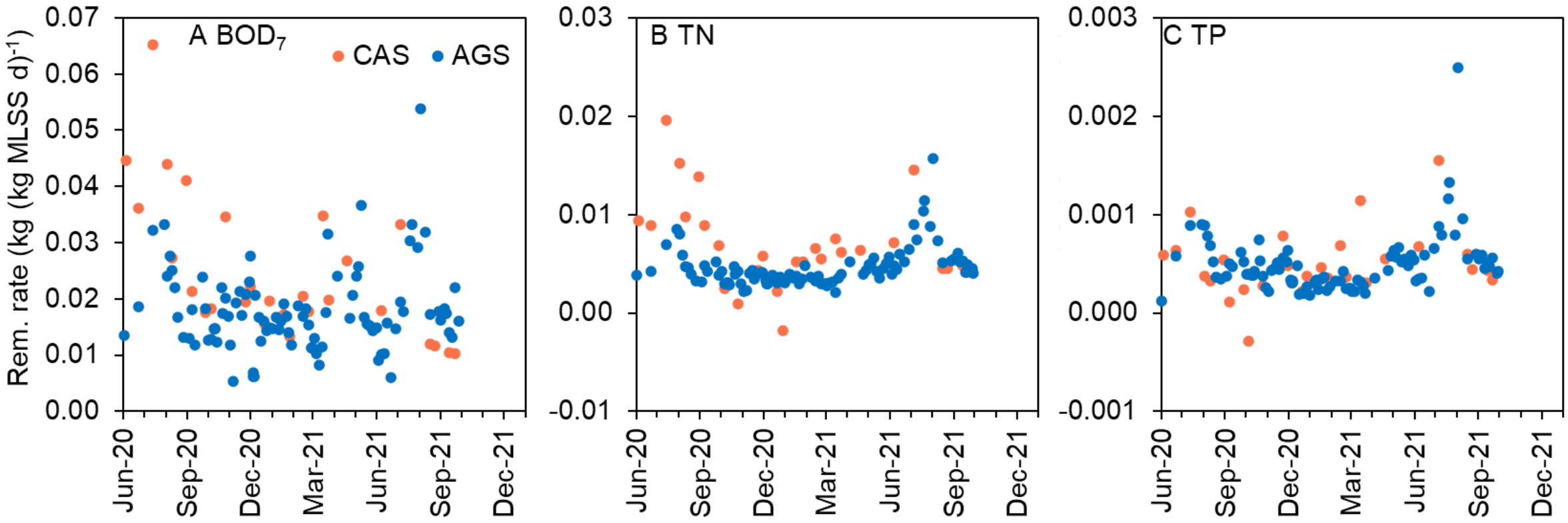

Figure S6. Biomass specific removal rates of A) BOD_7, B) TN, C) TP in the AGS and the CAS.

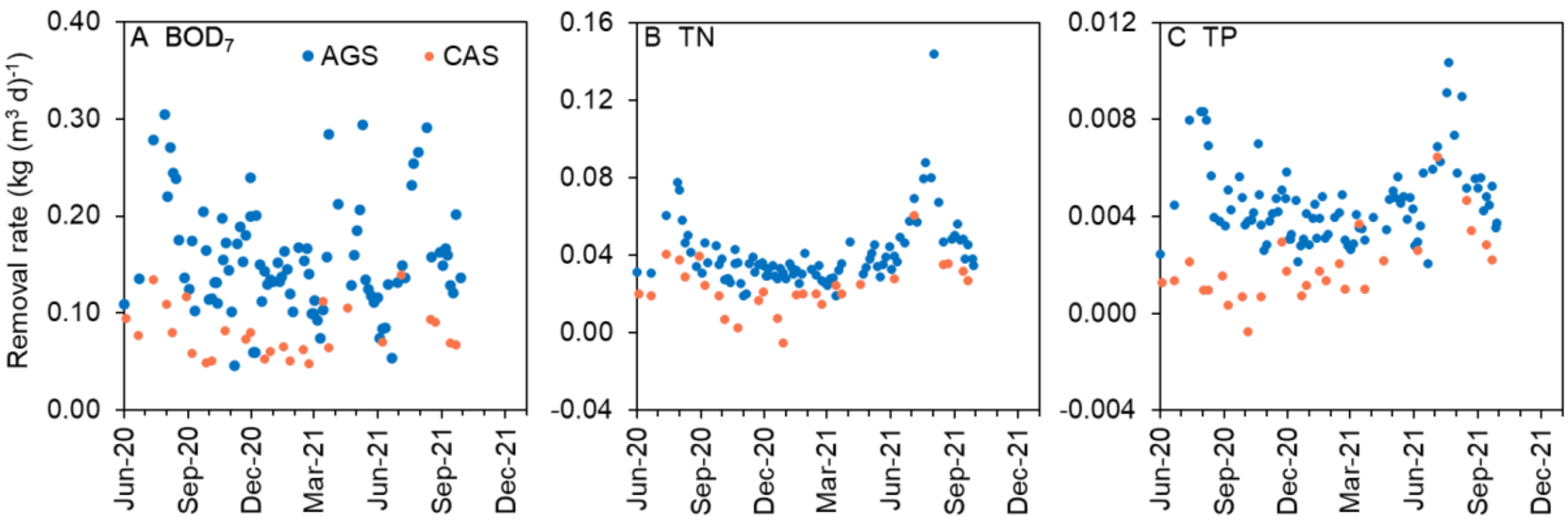

Figure S7. Volumetric removal rates of A) BOD_7, B) TN, C) TP in the AGS and the CAS.

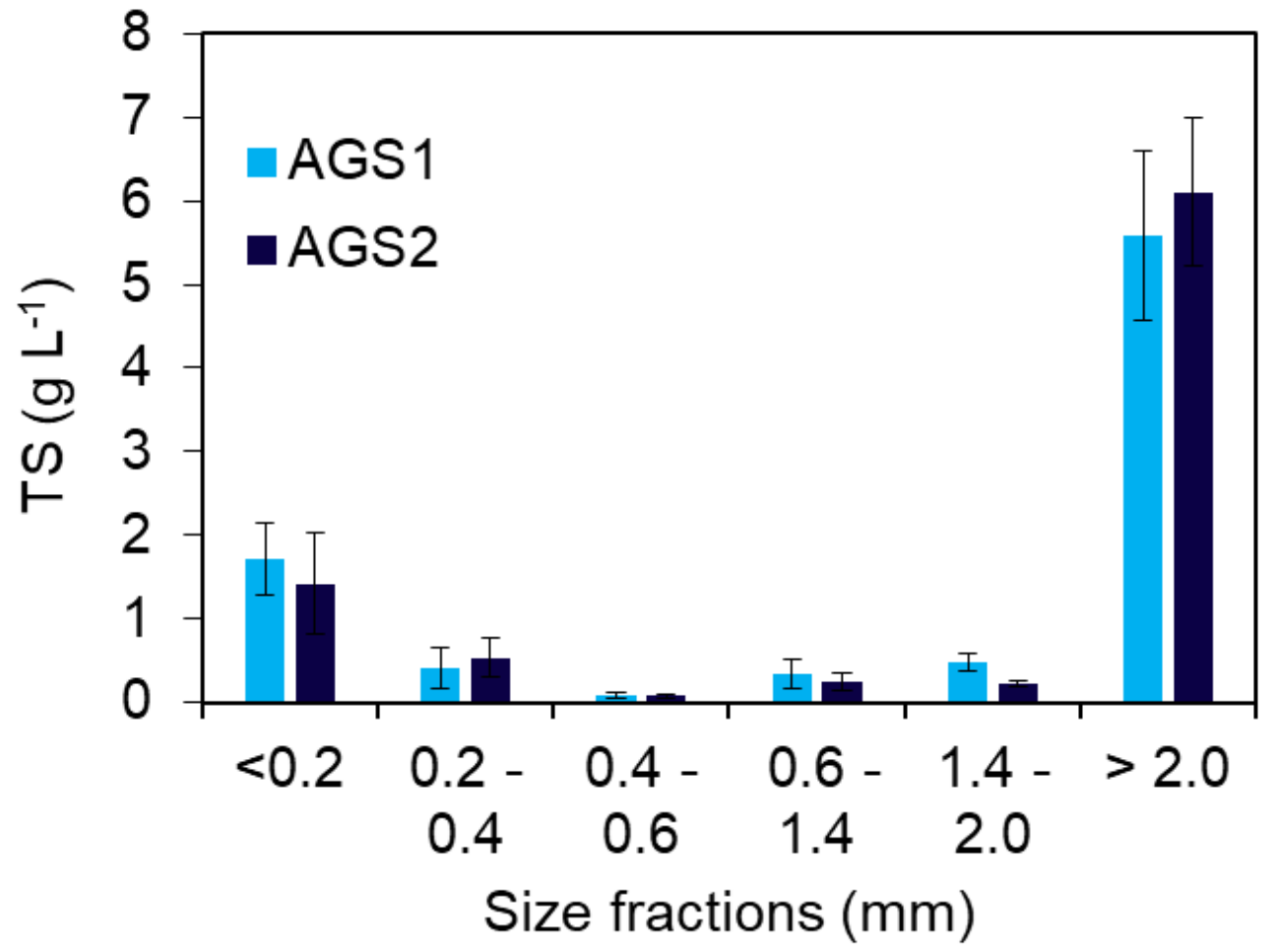

Figure S8. Aggregates average size distribution in AGS1 and AGS2.

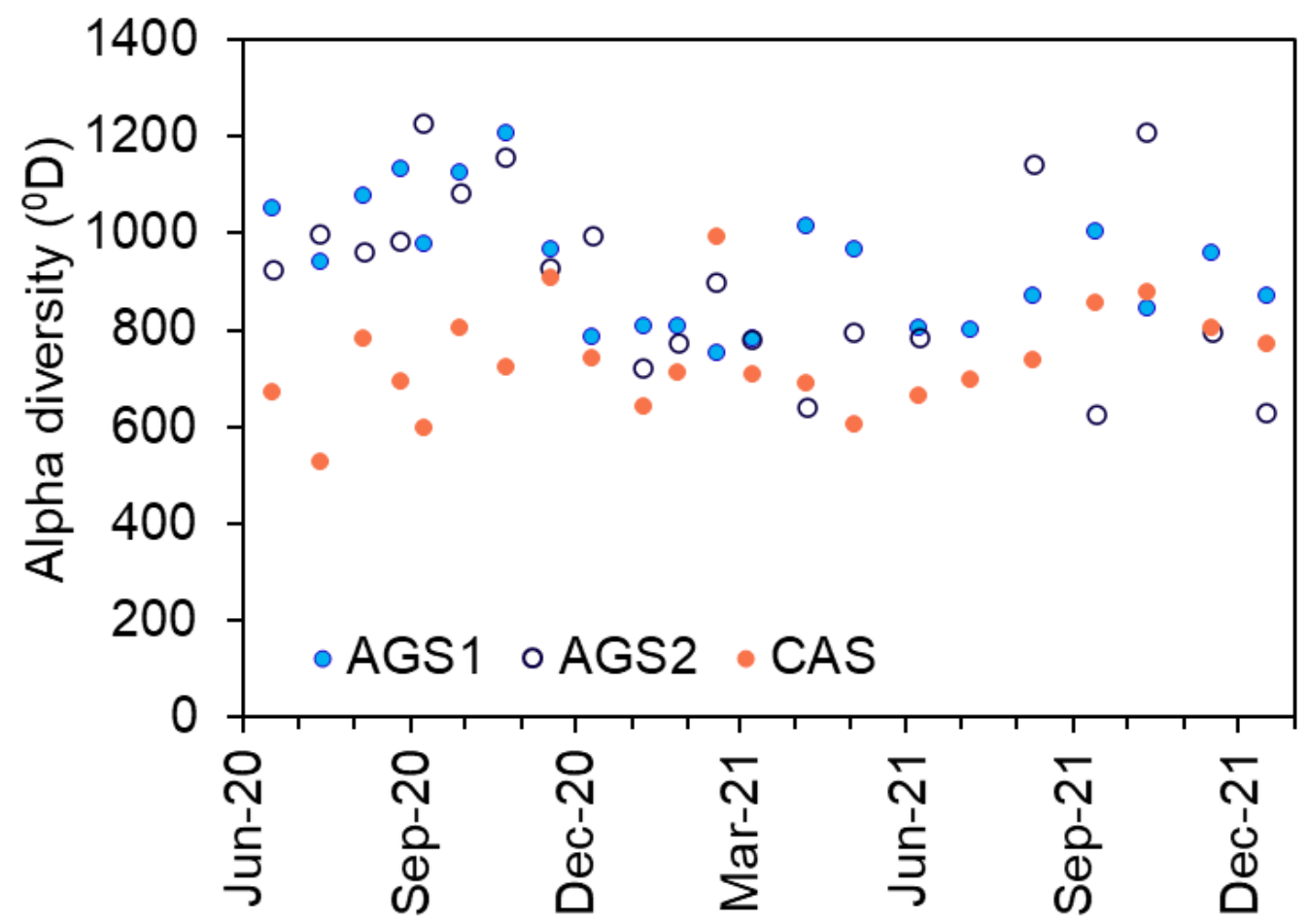

Figure S9. Alpha diversity over time (q=0).

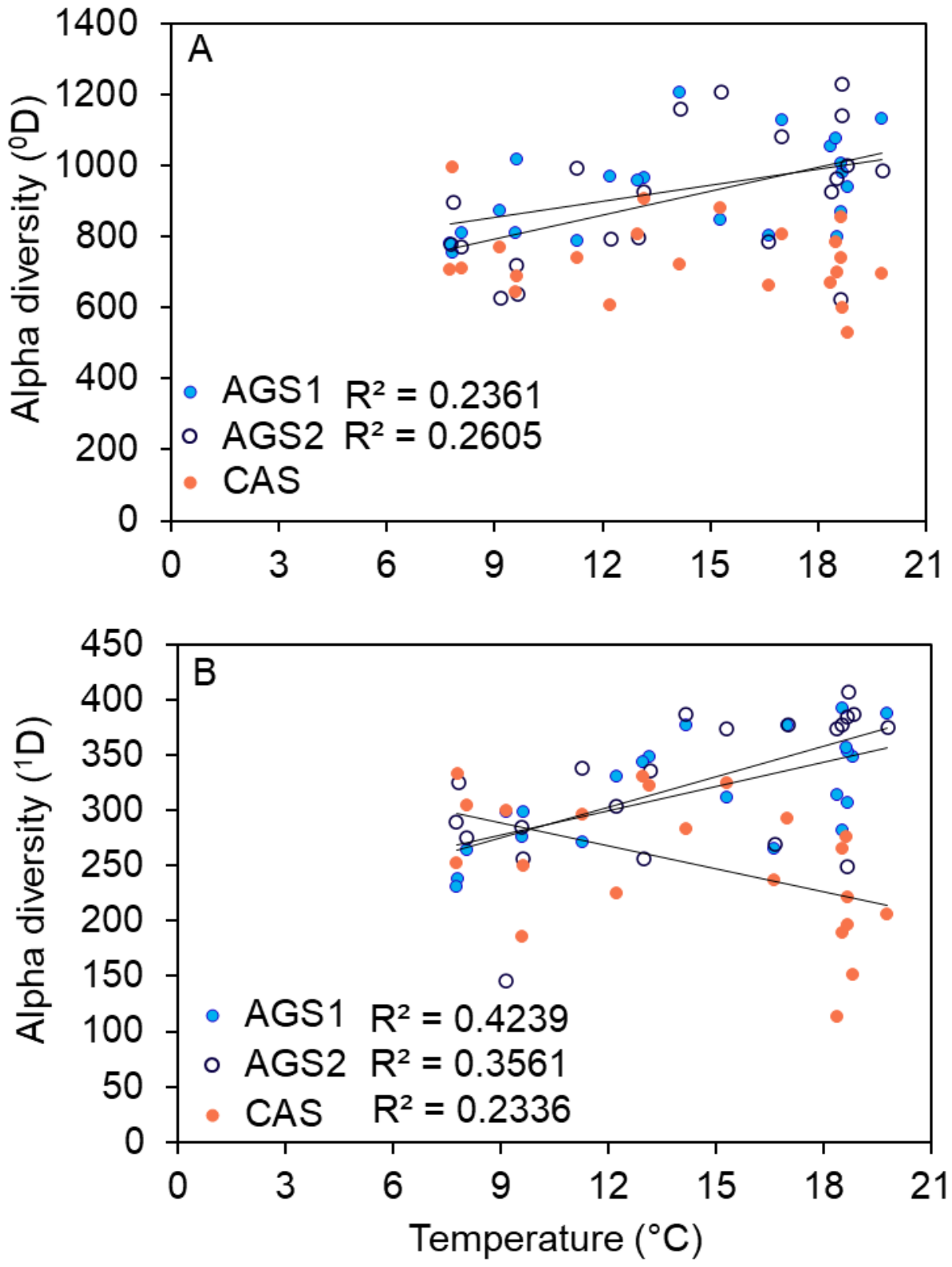

Figure S10. Alpha diversity for A) q=0 and B) q=1, plotted in relation to the temperature. R^2 is given for correlations with $p<0.05$.

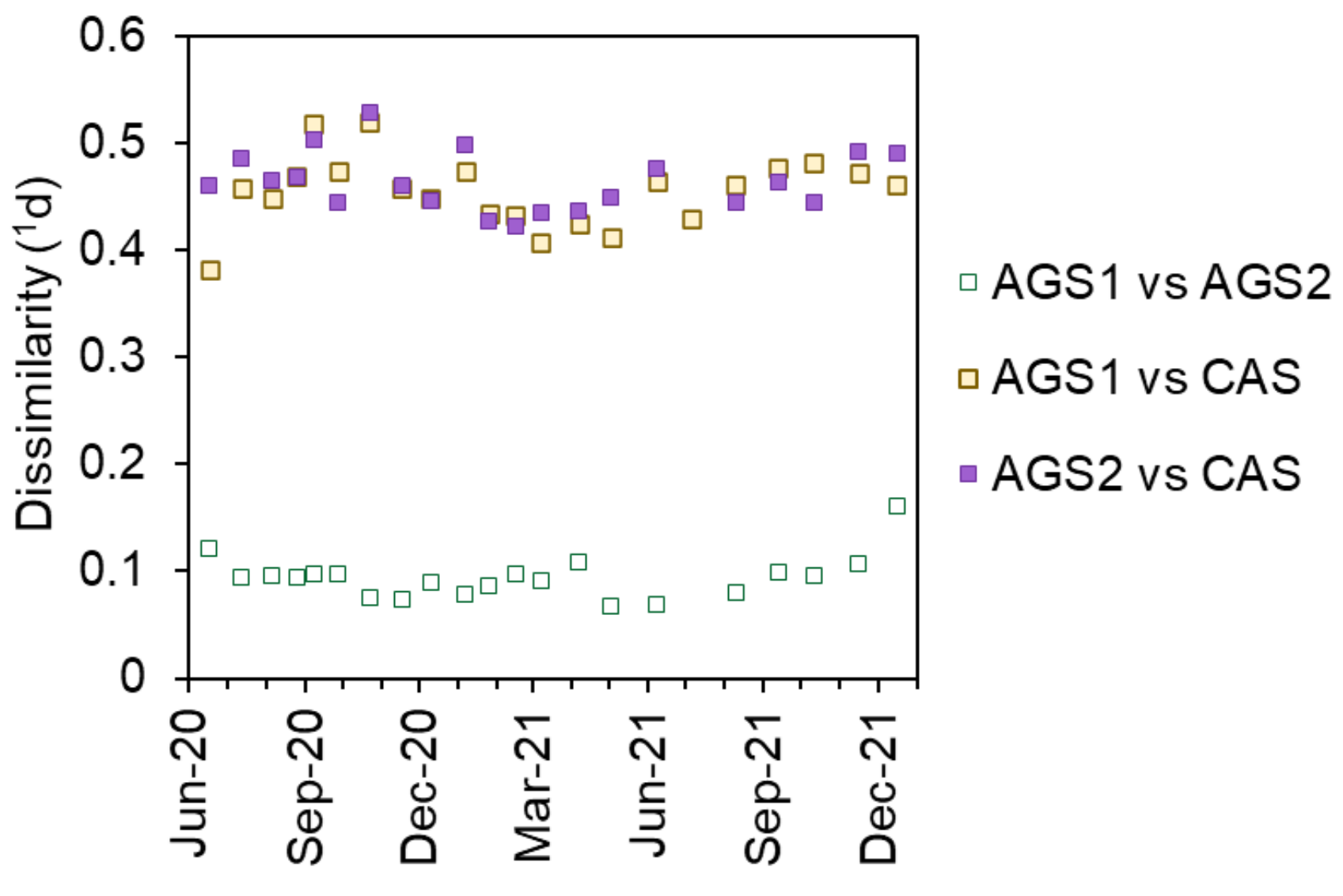

Figure S11. Dissimilarity (q=1) between samples taken the same day from the CAS and AGS1, and AGS2.

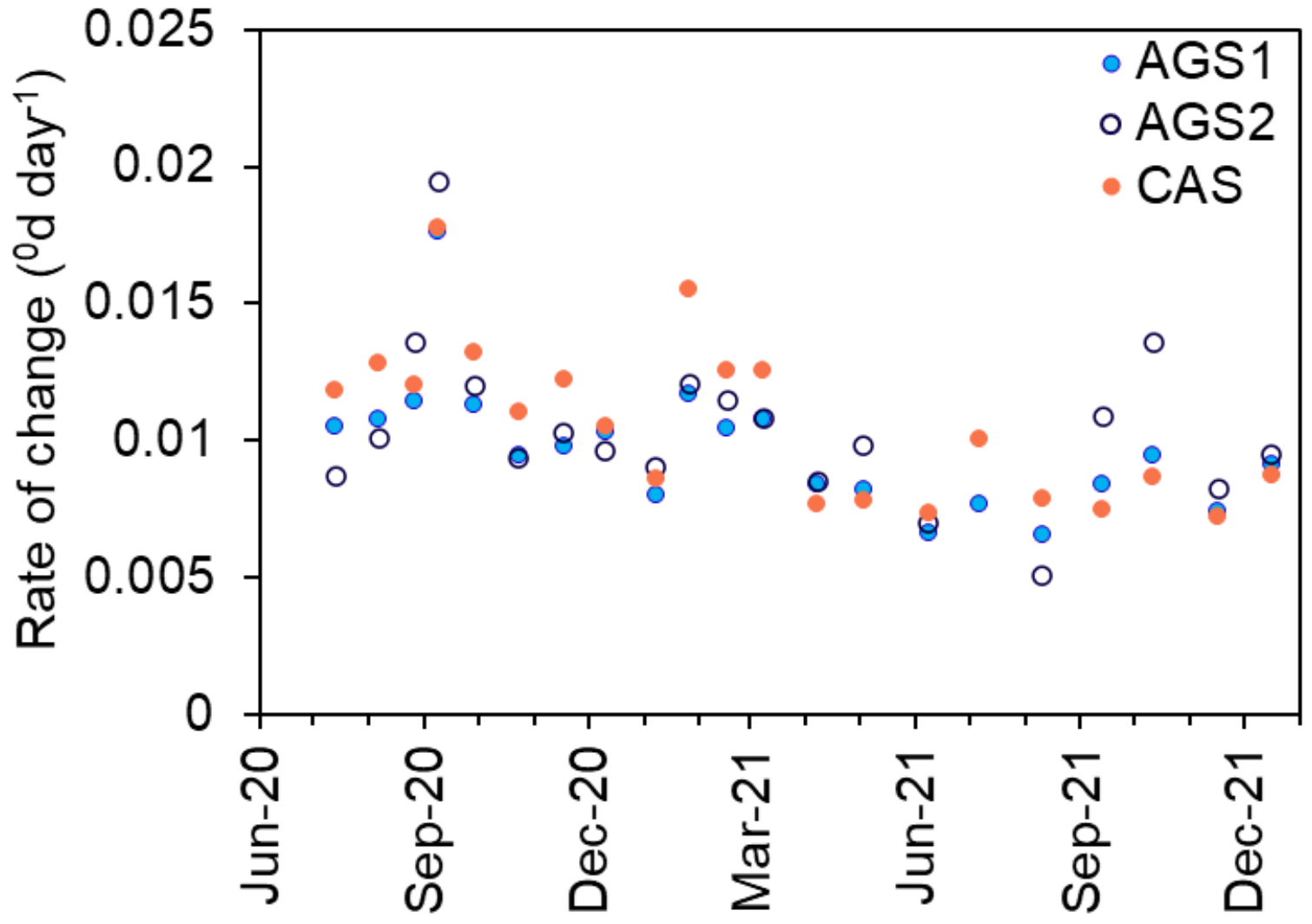

Figure S12. The rate of change in dissimilarity (q=0).

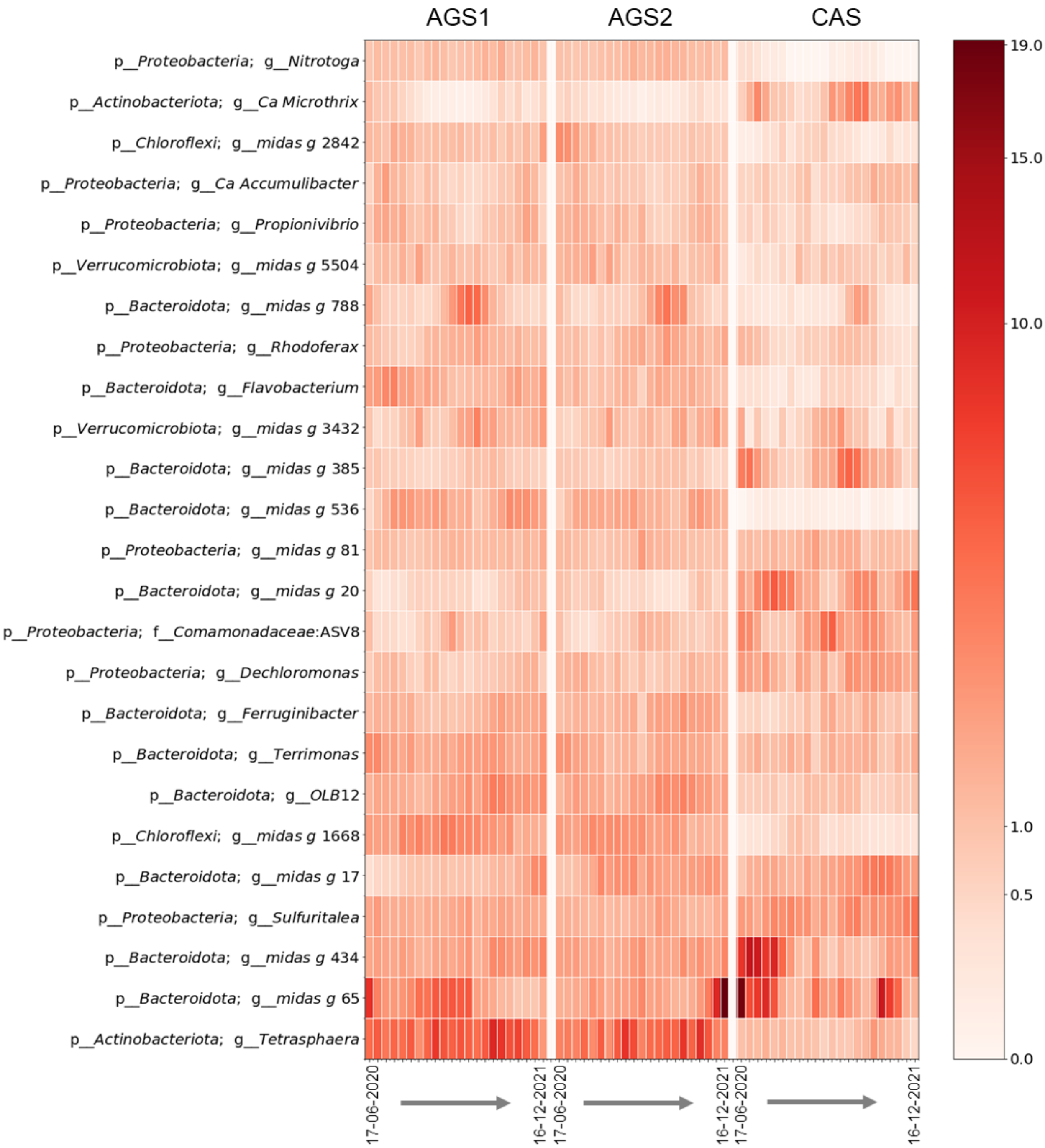

Figure S13. Heatmap of the 20 most abundant in genera AGS1, AGS2, and the CAS.

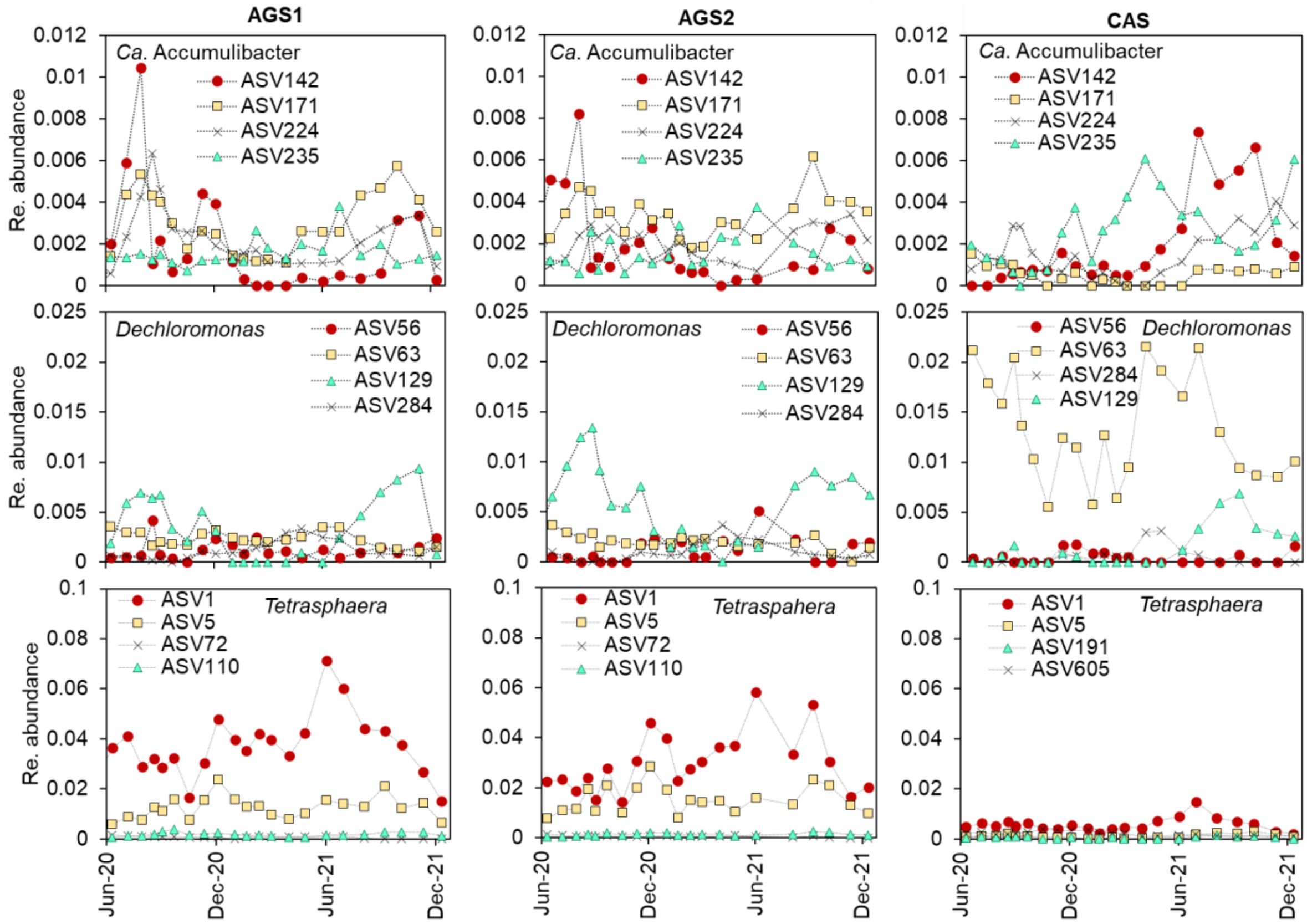

Figure S14. Relative abundance of *Ca.* Accumulibacter, *Dechloromonas* and *Tetrasphaera* in AGS1, AGS2 and CAS.

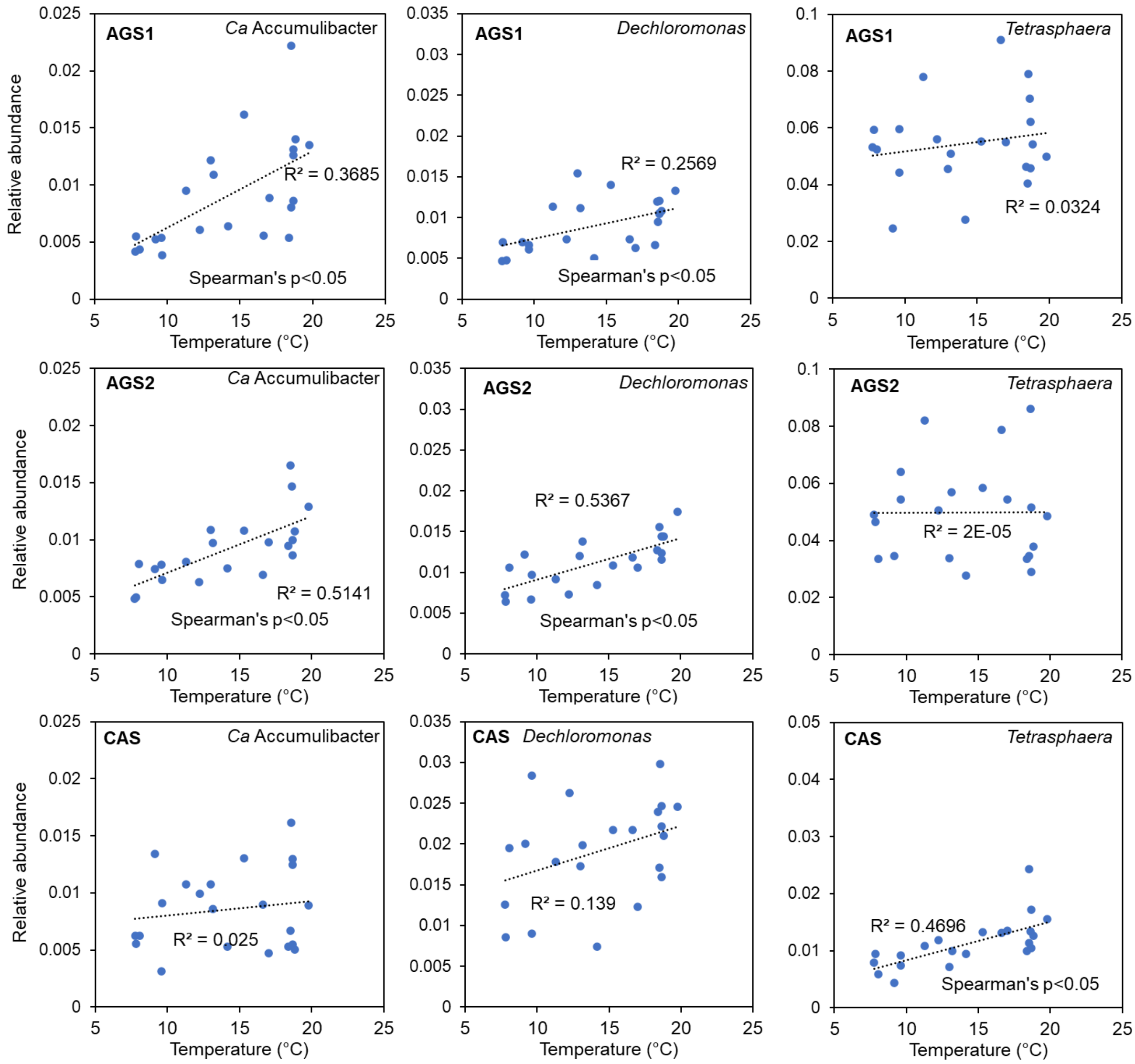

Figure S15. Plotted correlations of relative abundance and temperature for PAOs in AGS1, AGS2 and CAS. Significant correlations calculated with Spearman's correlation test are indicated for $p<0.05$ (See Table S5).

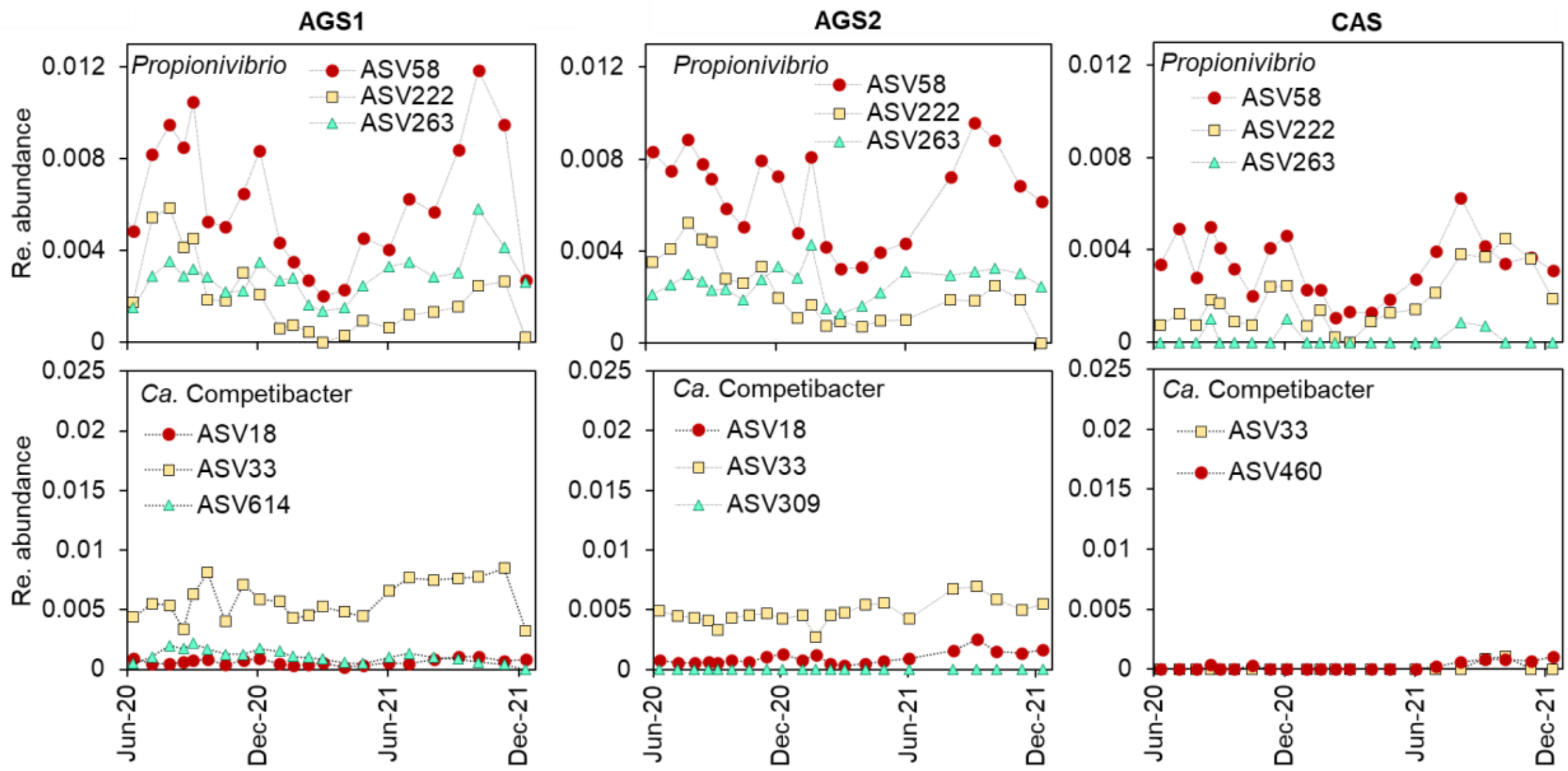

Figure S16. Relative abundance of *Propionivibrio* and *Ca.* Competibacter in AGS1, AGS2 and CAS. *One sample was not shown in this plot as the relative abundance was very high (0.1) compared with the other samples, for Ca. Competibacter in AGS2.

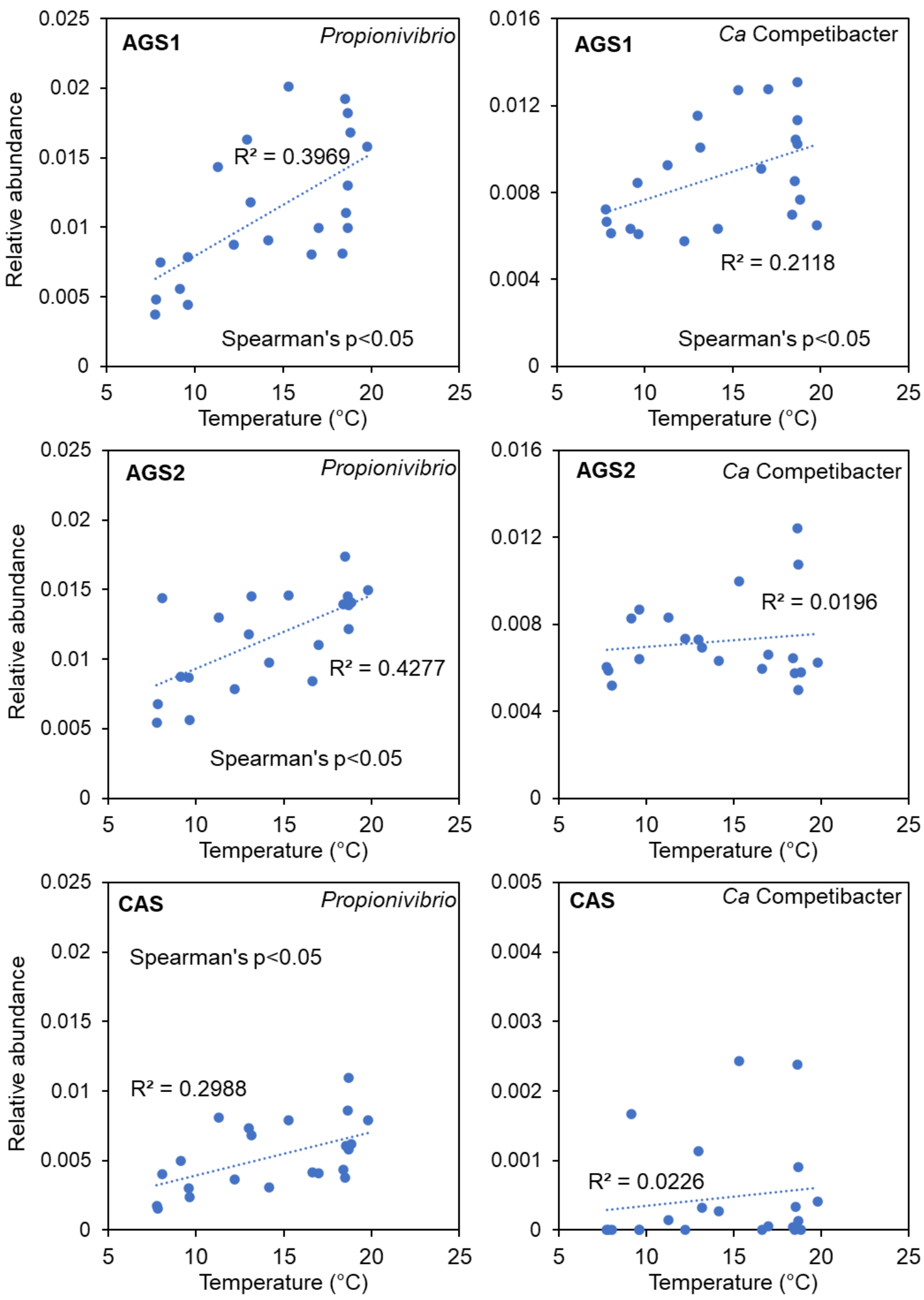

Figure S17. Plotted correlations of relative abundance and temperature for GAOs in AGS1, AGS2 and CAS. Significant correlations calculated with Spearman's correlation test are indicated for $p<0.05$ (See Table S5).

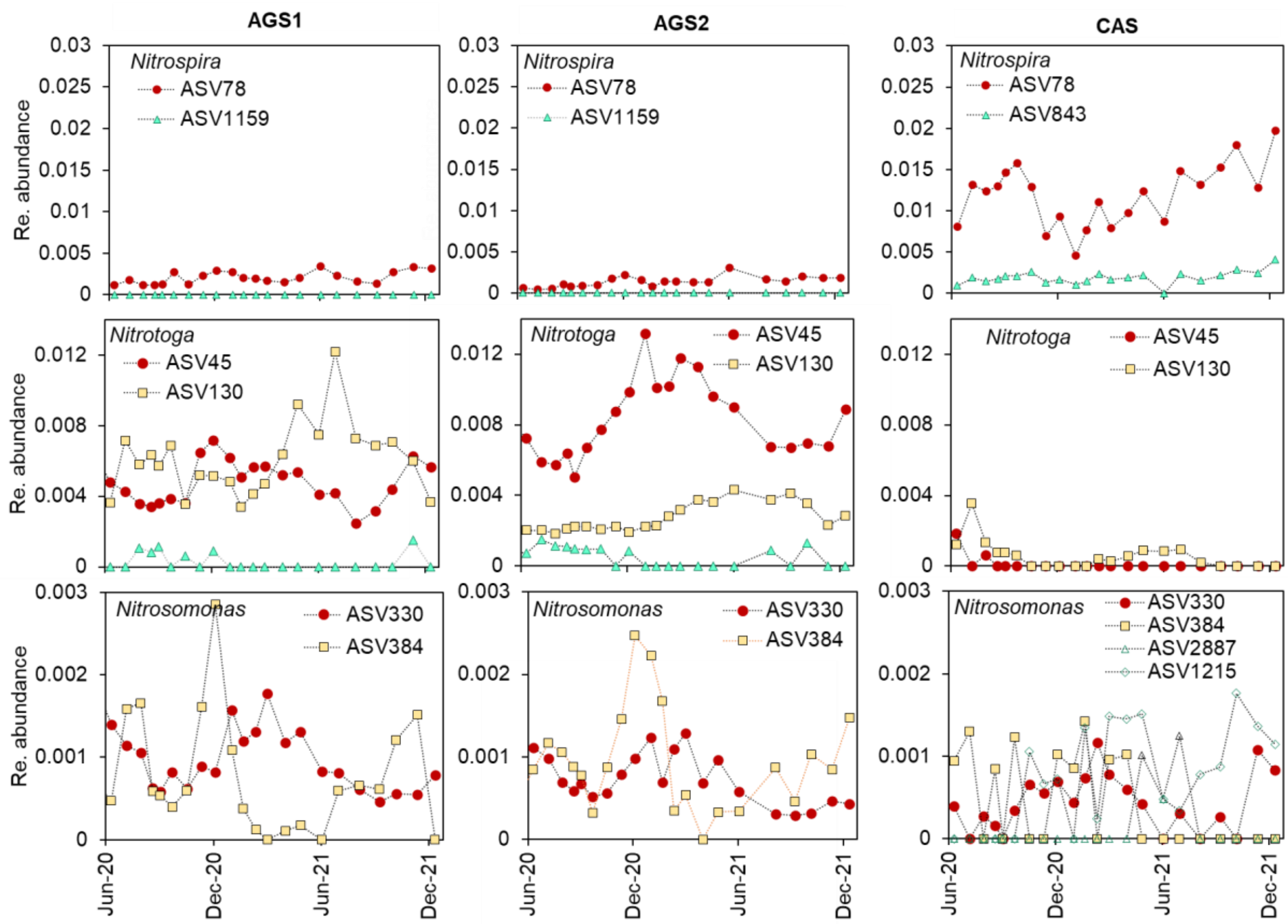

Figure S18. Relative abundance of *Nitrospira*, *Nitrotoga* and *Nitrosomonas* in AGS1, AGS2 and CAS.

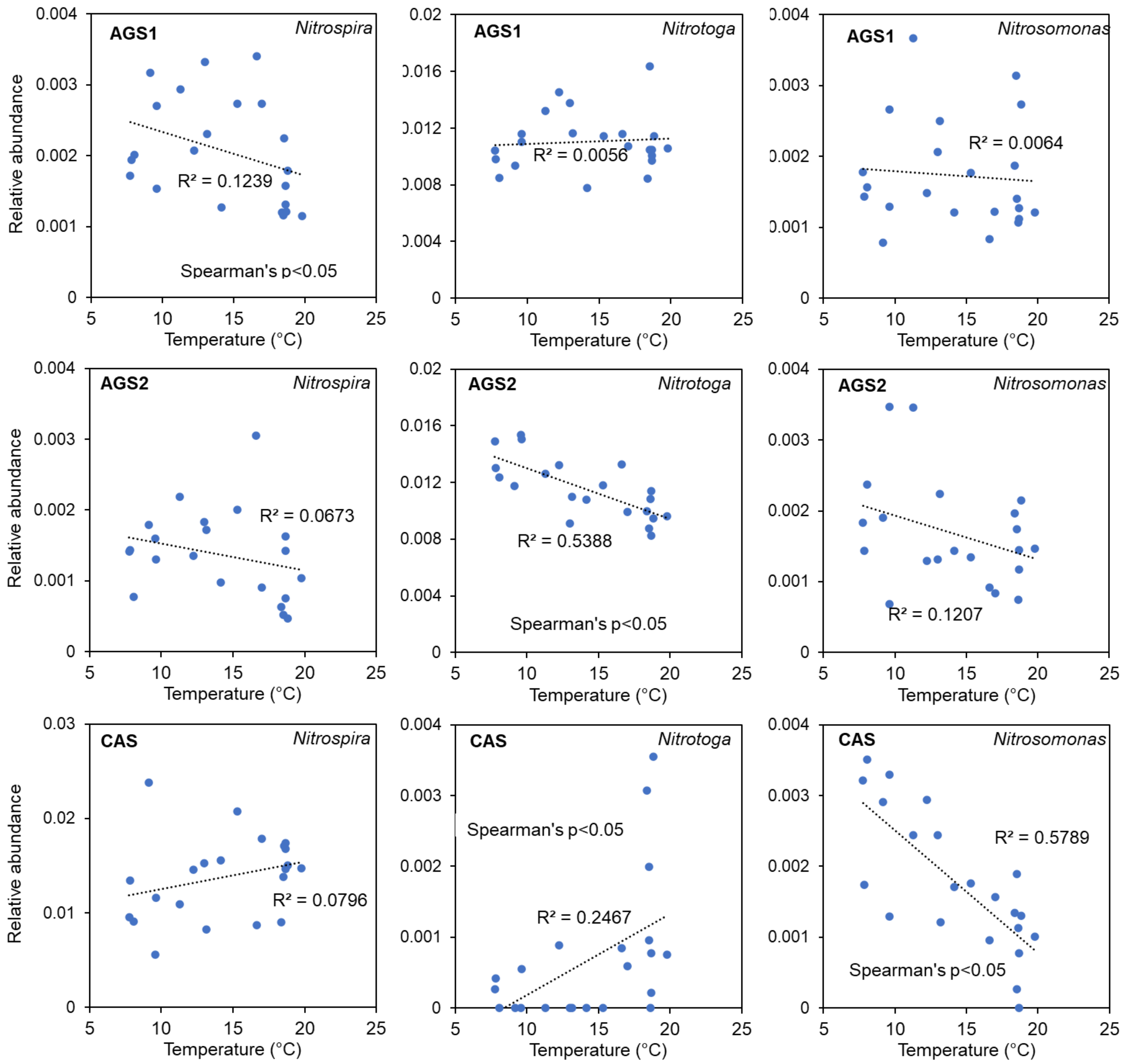

Figure S19. Plotted correlations of relative abundance and temperature for AOB and NOB in AGS1, AGS2 and CAS. Significant correlations calculated with Spearman's correlation test are indicated for $p<0.05$ (See Table S5).

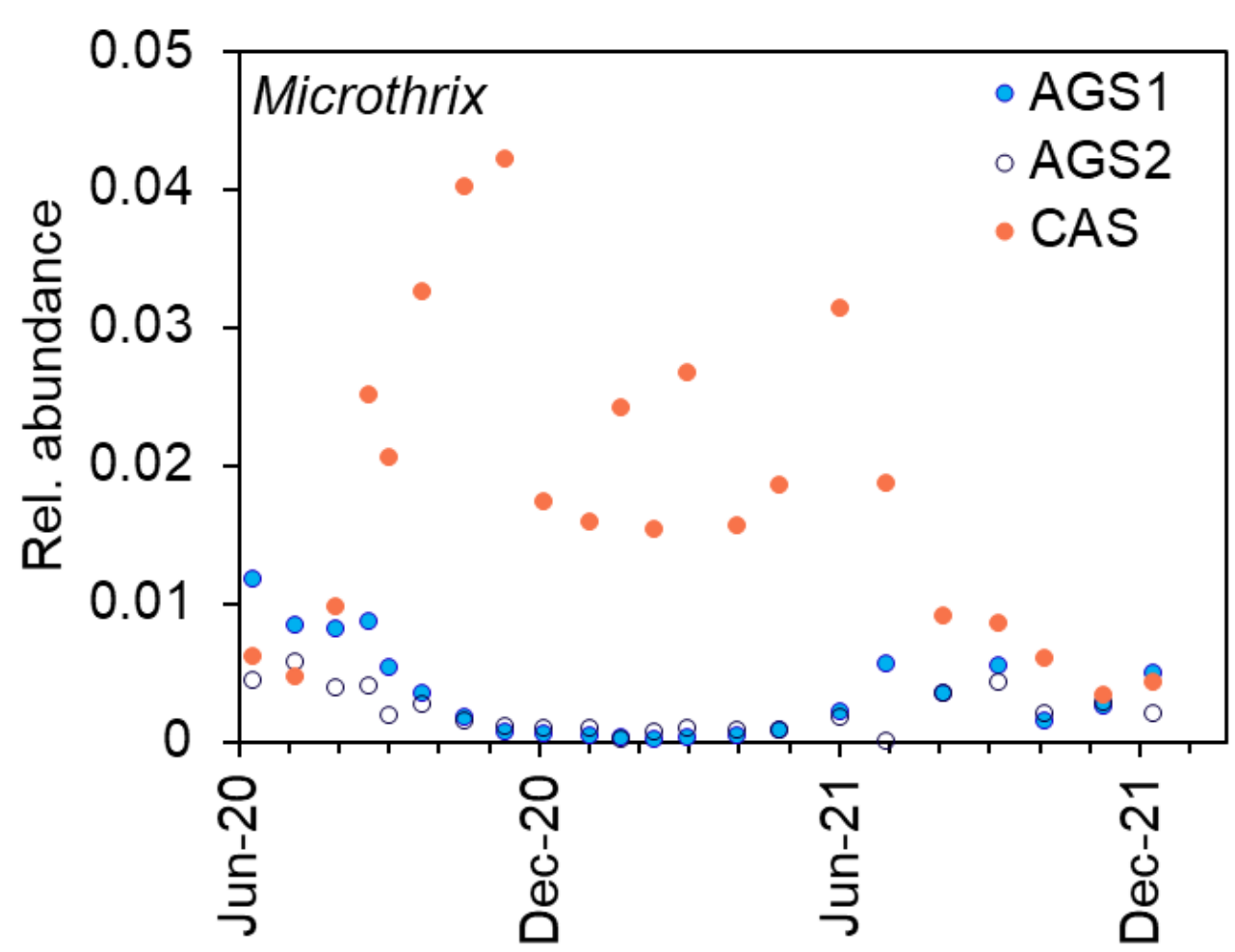

Figure S20. Relative abundance of *Microthrix* in the reactors.
